# Insight into Cellulose Dissolution with the Tetrabutylphosphonium Chloride–Water Mixture using Molecular Dynamics Simulations

**DOI:** 10.3390/polym12030627

**Published:** 2020-03-09

**Authors:** Brad Crawford, Ahmed E. Ismail

**Affiliations:** Department of Chemical and Biomedical Engineering, West Virginia University, Morgantown, WV 26505, USA

**Keywords:** water, biofuel, biomass, molecular dynamics simulation, ionic liquid, cellulose, diffusion, GLYCAM06

## Abstract

All-atom molecular dynamics simulations are utilized to determine the properties and mechanisms of cellulose dissolution using the ionic liquid tetrabutylphosphonium chloride (TBPCl)–water mixture, from 63.1 to 100 mol % water. The hydrogen bonding between small and large cellulose bundles with 18 and 88 strands, respectively, is compared for all concentrations. The Cl, TBP, and water enable cellulose dissolution by working together to form a cooperative mechanism capable of separating the cellulose strands from the bundle. The chloride anions initiate the cellulose breakup, and water assists in delaying the cellulose strand reformation; the TBP cation then more permanently separates the cellulose strands from the bundle. The chloride anion provides a net negative pairwise energy, offsetting the net positive pairwise energy of the peeling cellulose strand. The TBP–peeling cellulose strand has a uniquely favorable and potentially net negative pairwise energy contribution in the TBPCl–water solution, which may partially explain why it is capable of dissolving cellulose at moderate temperatures and high water concentrations. The cellulose dissolution declines rapidly with increasing water concentration as hydrogen bond lifetimes of the chloride–cellulose hydroxyl hydrogens fall below the cellulose’s largest intra-strand hydrogen bonding lifetime.

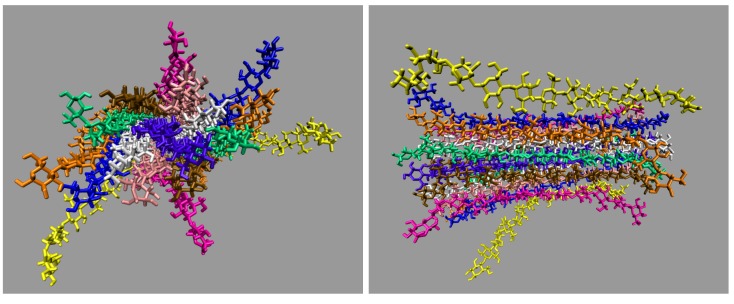



## 1. Introduction

The ability to economically convert biomass into an energy-dense fuel has the potential to offset the world’s fossil fuel consumption. If managed responsibly, biofuels can be a carbon-neutral source of energy by reconverting carbon dioxide back into biomass via photosynthesis. The crucial step toward this goal is the degradation of biomass into a product that can easily be converted into fuel, with limited energy input. By separating the biomass into individual cellulose strands, downstream processes can efficiently convert it into ethanol using enzymes or other catalyzed reactions. Ionic Liquids (ILs) are a class of solvents that are non-volatile, non-flammable, and thermally stable, with low melting points [1,2,3,4,5,6,7,8]. There are several ILs with the potential to dissolve cellulose, but many of them operate at higher temperatures and are ineffective even in low concentrations of water [1,5,6,7,9]. For cellulose dissolution to occur in an IL solution, the hydrogen bonds of a cellulose strand need to be broken [1,4]. In a 1-ethyl-3-methylimidazolium (EMIM) acetate (Ac) solution, Rabideau et al. found that breaking the inter-strand (between strands) hydrogen bonding of cellulose strands was insufficient for the cation to separate a cellulose strand from the rest of the cellulose bundle, and the intra-strand (within a strand) hydrogen bonding was the major barrier to cellulose strand separation since it inhibits the cellulose strand from twisting [4]. After the anion breaks the intra-strand hydrogen bonds allowing the strand to twist, the larger cations can wedge themselves under the cellulose strand separating it from the bundle, using its size to impede strand reformation [4]. The larger cations can physically separate the cellulose strand and prevent strand reformation, which small cations such as sodium are less capable of doing [1,4]. The cellulose dissolution also depends on how the polar and non-polar parts of the cellulose strand interact with the solvent, in many cases occurring between the cation and the peeling strand [1,4,10]. Higher temperatures can be required to dissolve cellulose, as the ILs typically have high viscosities in their pure form, and the hydrogen bonds and conformations of the hydroxymethyl groups of the cellulose bundle can change at elevated temperatures [1,10,11]. Water inhibits the cellulose dissolution in ILs by solvating the anion with increasing water concentration, leading to less sustained interaction with the cellulose bundle [1,2,3,4,5,6,7,8,12].

**Figure 1:**
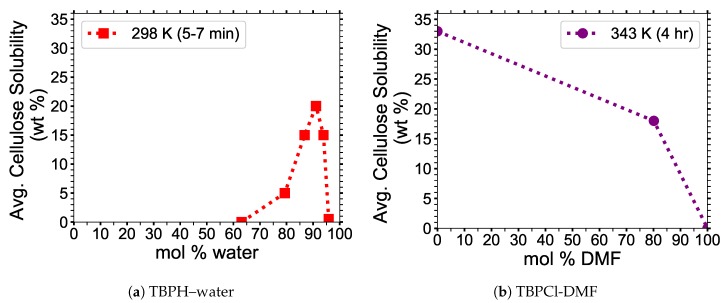
The experimental cellulose solubility in the tetrabutylphosphonium hydroxide (TBPH)–water and tetrabutylphosphonium chloride-dimethylformamide (TBPCl-DMF) solutions: (**a**) TBPH–water solution at 298K and 1 atm (Data from Abe et al.) [2]; (**b**) TBPCl-DMF solution at 343K and 1 atm (Data from Burns et al.) [3].

IL co-solvent mixtures have shown that co-solvents can increase the cellulose dissolution, creating a co-solvent concentration that maximizes the dissolvable amount of cellulose [13]. In pure ILs, the cation and anion are typically located close to each other due to the attractive Coulombic forces [12,13,14]. The co-solvent can surround both the cation and anion, further separating the cation and anion, which allows them to act individually and in sequence during the cellulose separation process [12,13,14]. Additionally, the co-solvent reduces viscosity and increases the diffusion of all the molecules in the solvent [1,12,13,14,15]. The decline of cellulose solubility at higher co-solvent concentrations is attributed to the loss in hydrogen bonding between the IL and the cellulose bundle (i.e., mostly the anion), which is also investigated in this study [13]. The degree of cellulose dissolution can significantly vary when changing the IL or co-solvent. For 1-allyl-3-methylimidazolium chloride (AMIMCl) in dimethylsulfoxide (DMSO) co-solvent, Zhang et al. have shown there exists a maximum cellulose dissolution between 0 to 50 mol % DMSO at 353 K [13]. Abe et al. have shown that tetrabutylphosphonium hydroxide (TBPH)–water mixtures have the ability to dissolve 20 wt % cellulose in 7 min at room temperature, at a concentration of 91.1 mol % water (see Figure 1a) [2]. The TBPH–water mixture has a working range for cellulose dissolution, between 86.8 to 93.9 mol % water [2]. Identifying the properties that allow the TBP class of ionic liquids to work in water at low to moderate temperatures could be a critical step in creating an economical biofuel. The tetrabutylphosphonium chloride (TBPCl)-dimethylformamide (DMF) co-solvent mixtures are capable of dissolving cellulose in high concentrations of DMF at 343 K (see Figure 1b). Since these data consist of only three data points, it is unclear if this solution has a dissolution maximum at a specific mol % DMSO. The above examples show that the imidazolium-based and the tetrabutylphosphonium-based cellulose dissolution maxima can be on opposite ends of the mol fraction of a co-solvent [2,13]. Cellulose dissolution appears to be determined by a combination of structural configurations, hydrogen bonding, diffusion regime, pairwise energy interactions, and other properties [4,12,13,16]. Therefore, each IL co-solvent mixture, or at least each cation class of IL/co-solvent, requires independent analysis.

Automating molecular dynamics simulations for the discovery of new ILs could produce superior IL–water combinations for cellulose dissolution in less time than traditional experimental testing alone. Using high concentrations of water as a co-solvent may be the most economical solution to cellulose dissolution since cellulose naturally contains up to 25 wt % water. Therefore, these new simulation-based search methods may be the key to designing an economically viable IL–water combination that can produce biofuels from waste biomass on an industrial scale. The pairwise energies, dissolution mechanisms, and hydrogen bonding can easily be studied via molecular dynamics simulations and compared to the existing imidazolium-based IL literature. The goal of this research is to understand the mechanisms and properties of TBPCl that allow cellulose dissolution at high water concentrations, which may enable future simulations to utilize machine learning algorithms to search for more economical and environmentally friendly ILs. Due to the toxic nature of TBPCl in its pure form and at low water concentrations, molecular dynamics simulations are an ideal way to study the TBPCl–water solution [17,18].

There are several different cellulose types defined by their molecular configurations and hydrogen bonding; for example, the cellulose Iα, Iβ, II, $III_{I}$, and other crystal phases. Previous work by Rabideau et al. showed that the Iβ crystal phase is more stable in the imidazolium-based IL solutions when compared to the Iα crystal phase, due to the differing molecular configurations and hydrogen bonding in the cellulose bundle [4]. The Iβ crystal phase is the most dominant in plant-based cellulose [19]. In this work, all-atom molecular dynamics simulations analyze the TBPCl–water solution’s interaction with an Iβ cellulose bundle, since the Iβ cellulose bundle is the most common in plant-based cellulose and it is harder to dissolve in IL solutions [4,19]. Hadden et al. showed that the GLYCAM06 cellulose model with the large Iβ cellulose bundles more accurately simulates the experimental properties of actual cellulose, which is demonstrated by evaluating the cellulose bundle twisting. The 81 strand bundle with 20 glycans matched the experimental twisting of the Iβ cellulose bundle within approximately one percent [19,20]. The smaller cellulose bundles twist more as the bundle size decreases [19,20]. Simulating the large cellulose bundles is very computationally expensive, and the simulations can only be conducted over a short timeframe. Therefore, this study compares the size effects between a small cellulose bundle and a large one [19,21], ensuring that the small cellulose bundle adequately exhibits the critical hydrogen bonding properties, as seen in the larger bundle. This comparison warrants the use of the small cellulose bundle for the main simulations, which allows a much longer time to study the cellulose dissolution process. The small and large cellulose bundles consisted of 18 and 88 cellulose strands, respectively. For the small cellulose bundle, the cellulose dissolution concentrations, mechanisms, strand separation distances, pairwise energies and hydrogen bonds between the solvent and the peeling or non-peeling cellulose strands, and the hydrogen bonding lifetimes of the solvent and the cellulose were determined. The paper begins with the simulation method and details. Next, the results of the small and large cellulose bundle comparison are presented, followed by the results of the small cellulose bundle’s dissolution properties and mechanisms. Finally, the summation of the analysis is presented in the discussion and conclusions sections. The highlights of the paper include the following: an estimated cellulose dissolution profile for the TBPCl–water solution; an evaluation of the critical hydrogen bond lifetimes of the anion–cellulose hydroxyl hydrogens with increasing water concentration (this could be a method of determining where cellulose dissolution is possible); an analysis of the TBP–peeling strand pairwise energies and the thermodynamic path to cellulose dissolution; and a detailed description of the cooperative cellulose dissolution mechanism utilized by the TBPCl–water solution is presented.

## 2. Simulation Methods and Details

Tetrabutylphosphonium chloride (TBPCl)–water mixtures were used to simulate the dissolution of an Iβ cellulose bundle via all-atom molecular dynamics. There are many different cellulose types defined by their molecular configurations, such as cellulose Iα, Iβ, II, $III_{I}$, and other crystal phases. In plants, cellulose occurs in both the Iα and Iβ crystal phases and is suspected to exist mostly in the Iβ crystal phase [19]. Both the Iα and Iβ crystal phases are known to be stable in water at low to moderate temperatures. The Iβ crystal phase appears to be more stable in ionic liquid (IL) solutions, due to its unique molecular configuration and hydrogen bonding within the cellulose bundle [4]. The Iβ crystal phase was selected since it is the most dominant in plant-based cellulose and due to its higher stability in ILs [4,19]. The simulations were performed using the 12-Dec-2018 version of the Large-scale Atomic/Molecular Massively Parallel Simulator (LAMMPS) software [22]. The visualizations, number of hydrogen bonds, and dihedral angles were generated from the Visual Molecular Dynamics (VMD) software [23]. The PACKMOL software was utilized to generate the initial packing configurations for the simulations [24], while the initial structure of the Iβ- cellulose bundle was built by means of the Cellulose-Builder code [25]. The large Iβ-cellulose bundle contains 88 individual strands and 24 glycans (24 glucose units) per strand, because it was previously determined that the 81-strand bundle with 20 glycans closely matched the experimental twisting data, with approximately one percent more cellulose bundle twisting in the simulations [19]. The small Iβ-cellulose bundle contains 18 individual strands and 12 glycans (12 glucose units) per strand. The small bundle size was selected because it was larger than a previous small bundle study, which had 10 strands and 8 glycans per strand [4], and this bundle size made these long simulations at various water concentrations computationally feasible. The TBPCl–water concentrations are based on experimental data from the tetrabutylphosphonium hydroxide (TBPH)–water system (see Figure 1 and Table 1 and Table 2) [2]. The TBPCl–water simulation boxes were designed to provide the cellulose with at least a 25 Å or greater distance through the periodic boundary condition. The solvent and cellulose dissolution concentrations are presented in mol % water and wt % dissolved cellulose, respectively (see Equations (1) and (2)).

(1) $$\mathrm{mol}\;\%\;\mathrm{water}=\frac{\left(\mathrm{molecules}\;\mathrm{of}\;\mathrm{water}\right)\left(100\right)}{\left(\mathrm{molecules}\;\mathrm{of}\;\mathrm{water}\right)+\left(\mathrm{molecules}\;\mathrm{of}\;\mathrm{TBPCl}\right)}$$

(2) $$\mathrm{wt}\;\%\;\mathrm{dissolved}\;\mathrm{cellulose}=\frac{\left(\mathrm{dissolved}\;\mathrm{cellulose}\;\mathrm{wt}\right)\left(100\right)}{\left(\mathrm{dissolved}\;\mathrm{cellulose}\;\mathrm{wt}\right)+\left(\mathrm{water}\;\mathrm{wt}\right)\left(\mathrm{TBPCl}\;\mathrm{wt}\right)}$$

The force field constants for the simulations were taken from the following sources: the TBP^+^ cation was from Zhou et al. [26]; the chloride anion was taken from Canongia Lopes et al., which derived an OPLS-AA/AMBER force field with the Lorentz–Berthelot mixing rules (note: these are also the same constants used for the OPLS-AA force field fitted to 68 unique ionic liquids by Sambasivarao et al.) [27,28,29,30]; the Glycosylation-dependent Cell Adhesion Molecule 2006 (GLYCAM06) force field was employed for the cellulose bundle [31]; and the three-site transferrable intermolecular potential (TIP3P)-pppm model was selected for water [32,33]. The force fields utilized for the TBPCl–water system were previously validated with the TIP4P water model [12,34]. Lorentz–Berthelot mixing rules were employed for mixing the force fields [29,30]. The potential energy equations are from the Assisted Model Building with Energy Refinement (AMBER) potential [35].

Timesteps of 2 fs were utilized with the velocity Verlet algorithm [36], extracting data every 10 ps. The short-range dispersion and electrostatic forces for all non-bonded atoms had cutoffs of 8 Å. The long-range electrostatics calculations used the Particle-Particle-Particle-Mesh (PPPM) method [37], with an accuracy of 10^−4^. The Isele–Holder method calculated the long-range dispersion forces [38], with a real space accuracy of 10^−3^ and a kspace accuracy of 2 × 10^−2^. These short-range cutoffs and long-range parameters were validated using smaller TBPCl–water simulations of approximately 10,000 atoms, with short-range dispersion and electrostatic force cutoffs of 10 Å for all non-bonded atoms. The simulations with the 10 Å cutoffs utilized more accurate long-range dispersion force calculations, with a real space accuracy and kspace accuracy of 10^−4^ and 2 × 10^−3^, respectively [38]. The TBPCl–water simulations with 10,000 atoms were also conducted using short-range dispersion and electrostatic force cutoffs of 8 Å for all non-bonded atoms, utilizing the same parameters in this study. All the simulations used the same PPPM accuracy. The production runs for this comparison were simulated using the *NPT* ensemble for 50 ns to get the density, and the *NVT* ensemble for 60 ns to calculate the chloride–water hydrogen bonding. The densities of the system and chloride–water hydrogen bonding between the 8 and 10 Å short-range cutoff simulations were compared at 60, 70, 90, and 99.97 mol % water. For the 60, 70, 90, and 99.97 mol % water concentrations, the density of the system varied between 0.01, 0.02, 0.15, and 0.12 percent, respectively, while the chloride–water hydrogen bonding varied between 2.7, 1.7, 0.9, and 0.6 percent, respectively. The short-range cutoff of 8 Å yielded variations that were within with the density and property deviations from the Isele–Holder method, and within a reasonable tolerance [38]. The short-range cutoffs of 8 Å reduced the computational cost of the simulations by approximately half, thereby allowing these long simulations to be conducted using the largest possible cellulose bundles at various water concentrations. The Isele–Holder method also theorized that the short-range cutoffs in many systems could be reduced while also providing accurate results, as long as the short-range cutoff was not smaller than twice the largest Lennard–Jones diameter, which agrees with these results [38]. The Nosé–Hoover system controlled the temperature and pressure with damping constants of 100 and 1000, respectively. These damping constants translate to relaxing the temperature and pressure every 100 and 1000 fs, respectively [12,39,40,41,42,43,44,45,46]. The simulations box size was modified isotropically with respect to the pressure damping. The SHAKE algorithm held the O–H bonds and angle of the water molecules rigid, along with any other covalent bonded hydrogens in the TBP or cellulose molecules [47]. The AMBER 1–4 interaction scaling factors from Cornell et al. were utilized for the TBP molecules [35], and the 1–4 interaction scaling factors of unity (i.e., 1) were applied for the cellulose’s GLYCAM06 force field [31].

Using the *NPT* ensemble, the TBPCl–water simulations were heated for 4 ns to 500 K, cooled to 360 K over 4 ns, and then allowed to equilibrate at 360 K for at least 3 ns. In order to insert the cellulose into the TBPCl–water system without overlapping atoms, all non-cellulose atoms were deleted if within 2 Å from the cellulose bundle [4]. Using the *NPT* ensemble, the TBPCl–water and cellulose simulations were started at 5 K and heated to 360 K over 1 ns, ensuring that the cellulose bundle remains in its most stable configuration. Once the TBPCl–water and cellulose system reached 360 K, the molecules were allowed to equilibrate for 4 ns, before moving the simulation to the *NVT* ensemble for the rest of the production simulations. A single simulation was conducted for each concentration.

LAMMPS does not support pairwise energy calculations when a system has mixed 1–4 interaction scaling factors or uses the Isele–Holder method for long-range dispersion forces [22,38]. The 1–4 interaction scaling factors must be zero to prevent unrealistic positive pairwise energies, since the bond, angle, and dihedral energies are not calculated in the same molecule, specifically the peeling cellulose strand. The bundle–peeling strand does calculate the pairwise energies from within its peeling strand provided they are 1–5 interactions or further, while the other analyses which are comparing differing molecules do not. The 1–2 and 1–3 interaction scaling factors were also zero, but this was the same as in all the simulations and re-runs. Therefore, the following calculation parameters were changed to compensate for this during the pairwise energies re-run data analysis: the pairwise energy calculations were set to zero for the 1–4 interaction scaling factors; the dispersion and electrostatic forces for all non-bonded atoms had cutoffs of 20 Å; no long-range dispersion forces were calculated; and the PPPM method was utilized for calculating long-range electrostatic forces [37]. The short-range dispersion force cutoffs were extended by 2.5 times (i.e., to 20 Å) to compensate for the removal of the long-range dispersion calculations; otherwise, the pairwise energies for the system could not be calculated. In many molecular dynamics simulations, the long-range dispersion forces are not used at all, with the standard short-range dispersion force cutoffs being around 10 to 12 Å.

**Figure 2:**
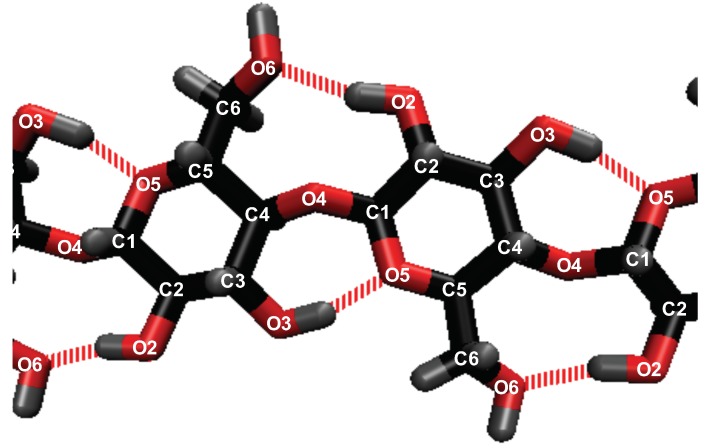
Atomic labeling for the cellulose strands. The black, red, and gray atoms are carbons, oxygens, and hydrogens, respectively. The dashed lines are hydrogen bonding within the cellulose strand or intra-strand hydrogen bonding. For clarity, only the carbons and the oxygens are labeled in the cellulose strands. In the cellulose strands: the hydrogens bonded to the oxygens are named HO2, HO3, and HO6, corresponding to the O2, O3, and O6 oxygens, respectively; the hydrogens bonded to the carbons are named H1, H2, H3, H4, H61, and H62, corresponding to the C1, C2, C3, C4, C6, and C6 carbons, respectively [23].

**Figure 3:**
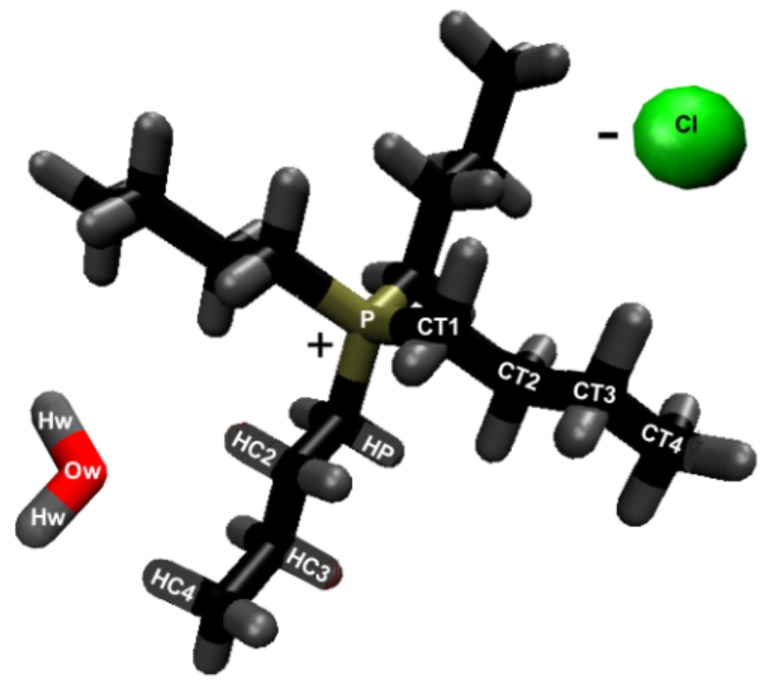
Atomic labeling for tetrabutylphosphonium (TBP), chloride (Cl), and water. The tan, black, red, and gray atoms are phosphorus, carbons, oxygens, and hydrogens, respectively. (**Left**) water molecule; (**center**) TBP molecule; (**right**) chloride molecule. For the TBP molecule, every atom was not labeled, but all butyl arms have the same symmetrical labeling. The right butyl arm is labeled with only the carbon atom labeling, while the bottom arm only has the hydrogen atoms labeled. The TBP atoms are labeled as follows: the CT’s are the carbons; the HP’s and HC’s are the hydrogens; the P is the phosphorus. The water’s atoms are Ow and Hw for oxygen and hydrogens, respectively. The chloride is labeled as Cl [23].

A hydrogen bond exists between the hydrogen acceptor and hydrogen if they are within 2.45 Å, and the hydrogen-donor–acceptor angle is 30 ${}^{\circ }$ or less [4,14,48,49,50,51,52,53,54,55]. The above conditions mandate that the donor–acceptor distance is 3.5 Å or less [4,14,48,49,50,51,52,53,54,55]. The number of hydrogen bonds was calculated using the VMD software. The calculated intra-strand (within a strand) hydrogen bonding in the cellulose does not include all the intra-strand bonding, and only includes the primary intra-strand bonding that is present in the experimental data (see Figure 2) [56]. The inter-strand (between strands) hydrogen bonding in the cellulose does represent all the hydrogen bonds between other cellulose strands. The atomic labeling for all the atoms in the system are represented in Figure 2 and Figure 3.

The hydrogen bonding lifetime calculations show the average bonding time between the atoms, which represents the average strength of the hydrogen bonds. The MD Analysis H-bond autocorrelation package generated the hydrogen bonding lifetimes, which were calculated in a series of four separate simulations with different time steps and run times [57,58,59]. Four simulations with varying time steps were utilized because it provided accurate hydrogen bonding lifetimes by minimizing the error in the time integration, especially in the case of the short hydrogen bonding lifetimes. All the additional simulations required to accurately calculate the hydrogen bonding lifetimes were started from the listed timepoints of the original simulations and stabilized for 0.2 ns before the data analysis began. The 10 ps time steps were taken from the original simulations. These four separate simulations were analyzed in the following order: a simulation with a time step of 0.01 ps and 100 ps run time; a simulation with a time step of 0.1 ps and 1000 ps run time; a simulation with a time step of 1 ps and 10,000 ps run time; and finally the original simulation time step of 10 ps with a run time that goes to the end of the simulation. The simulations were analyzed in order until one of the simulations provided a hydrogen bonding auto-correlation value of zero (the maximum value is 1). If a simulation finished without its auto-correlation value reaching zero, then the analysis continued by moving on to the next simulation in this series. Once a simulation in this series provided a zero value for the auto-correlation function, the current value of the hydrogen bonding lifetime was selected for the hydrogen bonding pair. If the auto-correlation function never reached zero even after the end of the last simulation in the series, the data were recorded, and the hydrogen bonding lifetime is presented with a “greater than” symbol (>). All the presented hydrogen bonding lifetimes were averaged over 10 samples, with each sample using 80% of the runtime for its analysis.

### 2.1. Simulations of the Small Iβ Cellulose Bundle (18 Strands)

**Figure 4:**
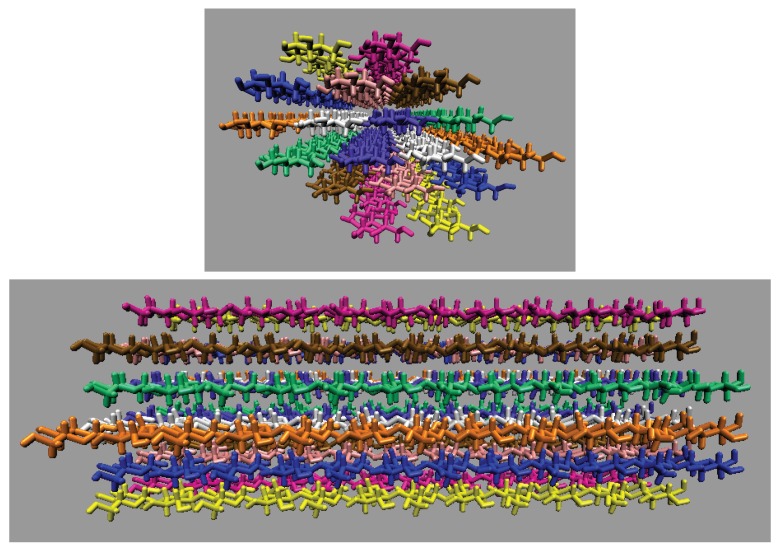
The initial configuration of the small Iβ cellulose bundle constructed via Cellulose-Builder [25]. The cellulose bundle has 18 individual strands with 12 glycan units per strand. The mirror image cellulose strands are identified using the same color [23].

After equilibration, the production runs were conducted using the *NVT* ensemble for the remaining 600 ns. The smaller cellulose strands were simulated for a much longer time, as they required less computational power. Table 1 shows a detailed breakdown of the molecules in the simulation, and Figure 4 shows the initial configuration of the small cellulose bundle. The small cellulose bundle contains 12 glycans per strand. The simulations have final box dimensions of 99 to 105 Å in all axis directions.

### 2.2. Simulations of the Large Iβ Cellulose Bundle (88 Strands)

**Figure 5:**
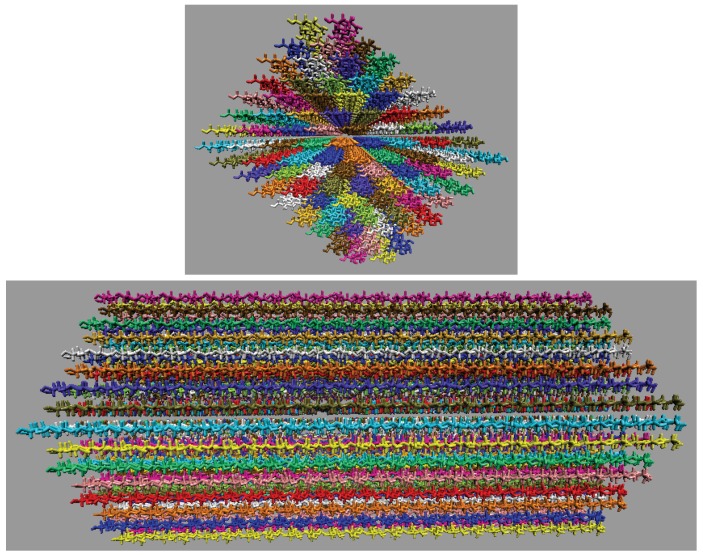
The initial configuration of the large Iβ cellulose bundle constructed via Cellulose-Builder [25]. The cellulose bundle has 88 individual strands with 24 glycan units per strand. The mirror image cellulose strands are identified using the same color [23].

After equilibration, the production runs were conducted for the remaining 20 ns using the *NVT* ensemble. The duration of the simulations for the 88 strand bundle was short due to the computational cost of these large systems. Table 2 details the molecular breakdown of the simulations, and Figure 5 shows the initial configuration of the large cellulose bundle. The large cellulose bundle contains 24 glycans per strand. The simulations have final box dimensions of 185 to 191Å in all axis directions.

## 3. Results: Small and Large Iβ Cellulose Bundle Comparison

**Figure 6:**
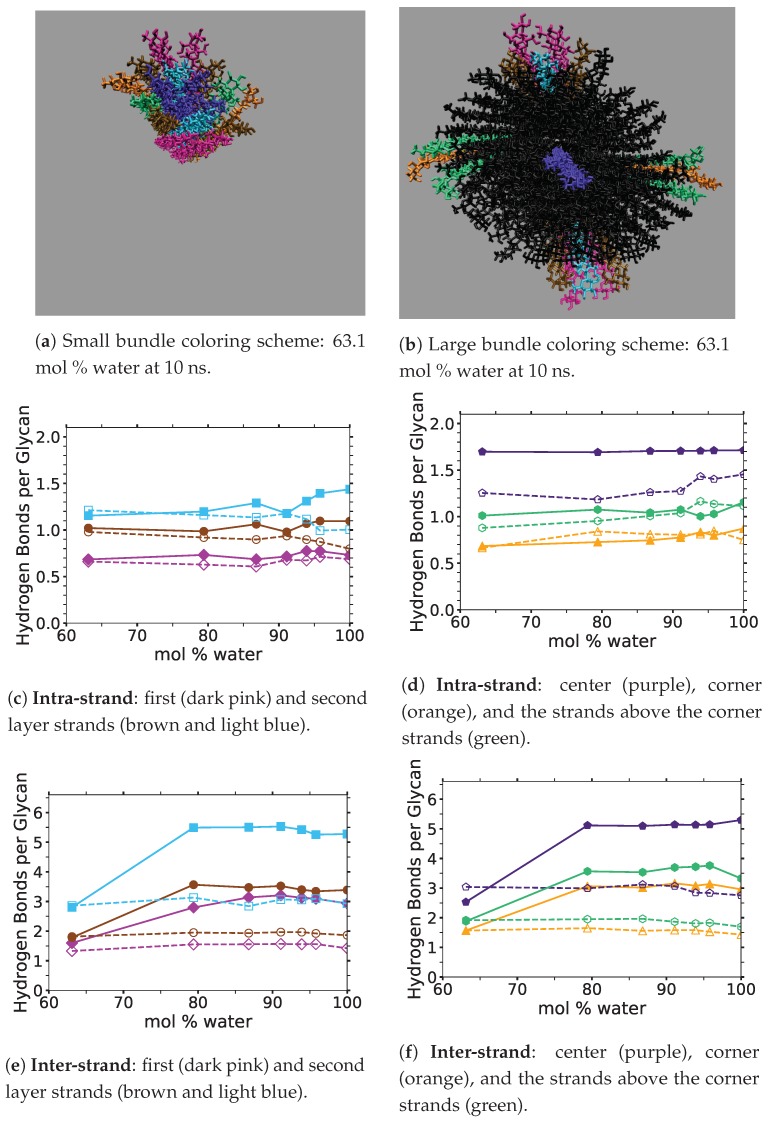
Average inter-stand and intra-strand hydrogen bonding comparison between the small and large cellulose bundles at various water concentrations: (**a**) small bundle coloring scheme; (**b**) large bundle coloring scheme; (**c**) the intra-strand hydrogen bonds for the first and second layers; (**d**) the intra-strand hydrogen bonds for the center, corner, and the strands above the corner strands; (**e**) the inter-strand hydrogen bonds for the first and second layers; (**f**) the inter-strand hydrogen bonds for the center, corner, and the strands above the corner strands. All data points were averaged from 10 to 20 ns. The solid lines and filled markers represent the large cellulose bundle, and the dashed lines and unfilled markers represent the small cellulose bundle. The colored strands are matched and compared between the plots for the small and large bundles and the images in this figure to identify similar hydrogen bonding behavior based on their locations (see Figure 6a,b for the color-coded images). The black cellulose strands are not compared. The small and large cellulose bundle intra-strand hydrogen bonding is based on 11 glycans and 23 glycans, respectively, as the last glycan has no potential bonding partner.

**Figure 7:**
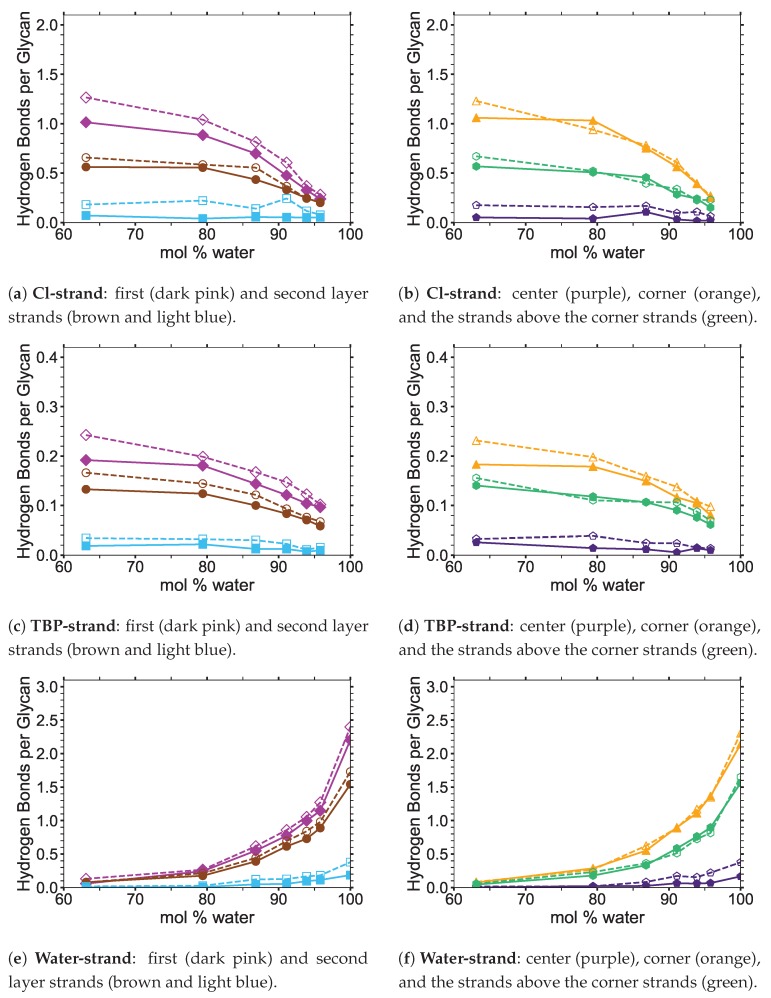
Average solvent hydrogen bonding comparison between the small and large cellulose bundles at various water concentrations: (**a**) the Cl-strand hydrogen bonds for the first and second layers; (**b**) the Cl-strand hydrogen bonds for the center, corner, and the strands above the corner strands; (**c**) the TBP-strand hydrogen bonds for the first and second layers; (**d**) the TBP-strand hydrogen bonds for the center, corner, and the strands above the corner strands; (**e**) the water-strand hydrogen bonds for the first and second layers; (**f**) the water-strand hydrogen bonds for the center, corner, and the strands above the corner strands. All data points were averaged from 10 to 20 ns. The solid lines and filled markers represent the large cellulose bundle, and the dashed lines and unfilled markers represent the small cellulose bundle. The colored strands are matched and compared between the plots for the small and large bundles and the images to identify similar hydrogen bonding behavior based on their locations (see Figure 6a,b for the color-coded images). The black cellulose strands are not compared.

**Figure A1:**
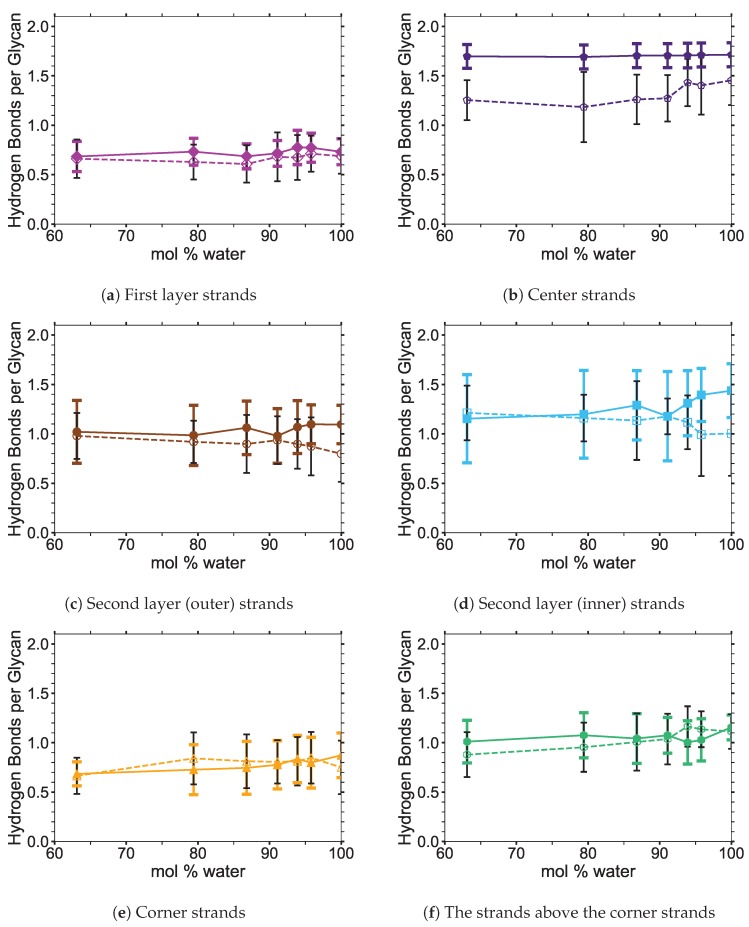
Average **intra-strand** hydrogen bonding comparison with standard deviations between the small and large cellulose bundles at various water concentrations: (**a**) the first layer strands; (**b**) the center strands; (**c**) the second layer (outer) strands; (**d**) the second layer (inner) strands; (**e**) the corner strands; (**f**) the strands above the corner strands. All data points were averaged from 10 to 20 ns. The solid lines, filled markers, and colored error bars represent the large cellulose bundle, and the dashed lines, unfilled marker, and black error bars show the small cellulose bundle. The colored strands are matched and compared between the plots for the small and large bundles and the images to identify similar hydrogen bonding behavior based on their locations (see Figure 6a,b for the color-coded images). The black cellulose strands are not compared. The small and large cellulose bundle intra-strand hydrogen bonding is based on 11 glycans and 23 glycans, as the last glycan has no potential bonding partner.

**Figure A2:**
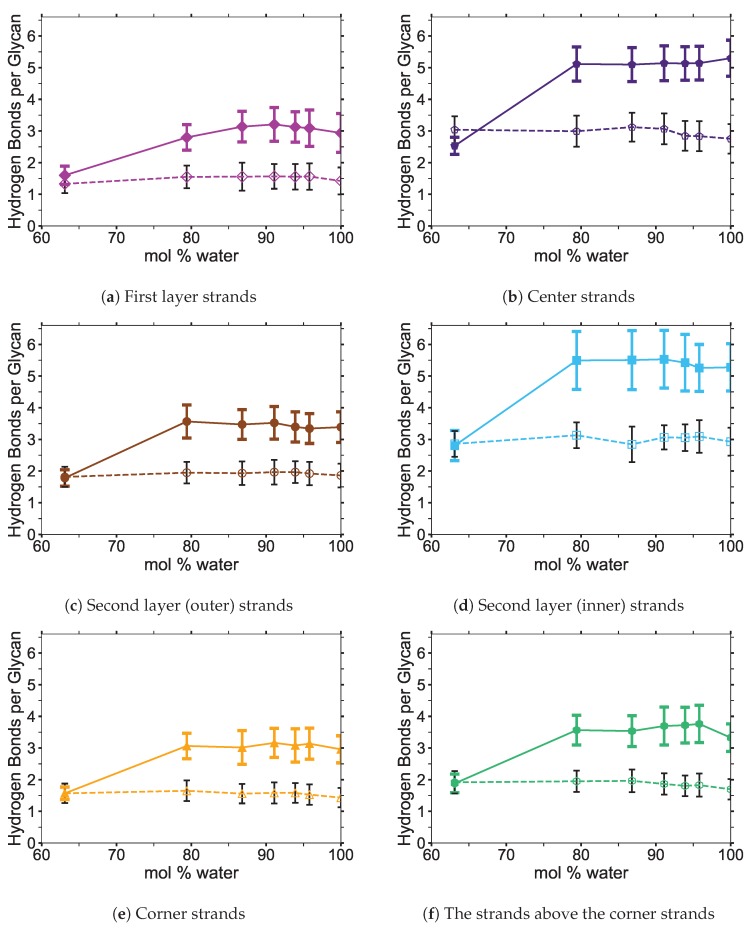
Average **inter-strand** hydrogen bonding comparison with standard deviations betweenthe small and large cellulose bundles at various water concentrations: (**a**) the first layer strands; (**b**) the center strands; (**c**) the second layer (outer) strands; (**d**) the second layer (inner) strands; (**e**) the corner strands; (**f**) the strands above the corner strands. All data points were averaged from 10 to 20 ns. The solid lines, filled markers, and colored error bars represent the large cellulose bundle, and the dashed lines, unfilled marker, and black error bars show the small cellulose bundle. The colored strands are matched and compared between the plots for the small and large bundles and the images to identify similar hydrogen bonding behavior based on their locations (see Figure 6a,b for the color-coded images). The black cellulose strands are not compared.

**Figure A3:**
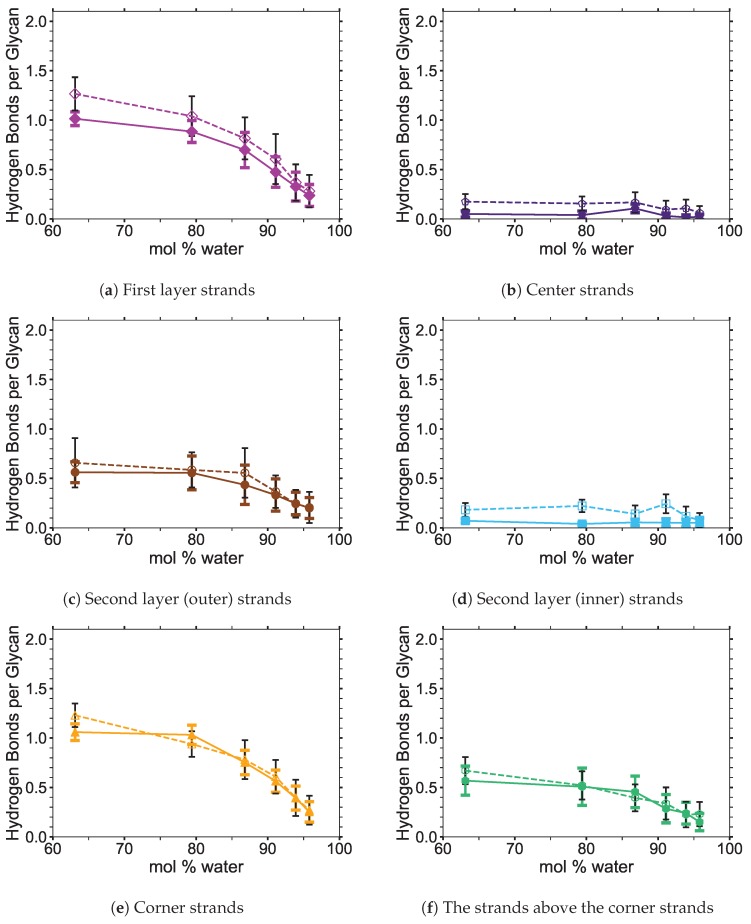
Average **Cl-strand** hydrogen bonding comparison with standard deviations between the small and large cellulose bundles at various water concentrations: (**a**) the first layer strands; (**b**) the center strands; (**c**) the second layer (outer) strands; (**d**) the second layer (inner) strands; (**e**) the corner strands; (**f**) the strands above the corner strands. All data points were averaged from 10 to 20 ns. The solid lines, filled markers, and colored error bars represent the large cellulose bundle, and the dashed lines, unfilled marker, and black error bars show the small cellulose bundle. The colored strands are matched and compared between the plots for the small and large bundles and the images to identify similar hydrogen bonding behavior based on their locations (see Figure 6a,b for the color-coded images). The black cellulose strands are not compared.

**Figure A4:**
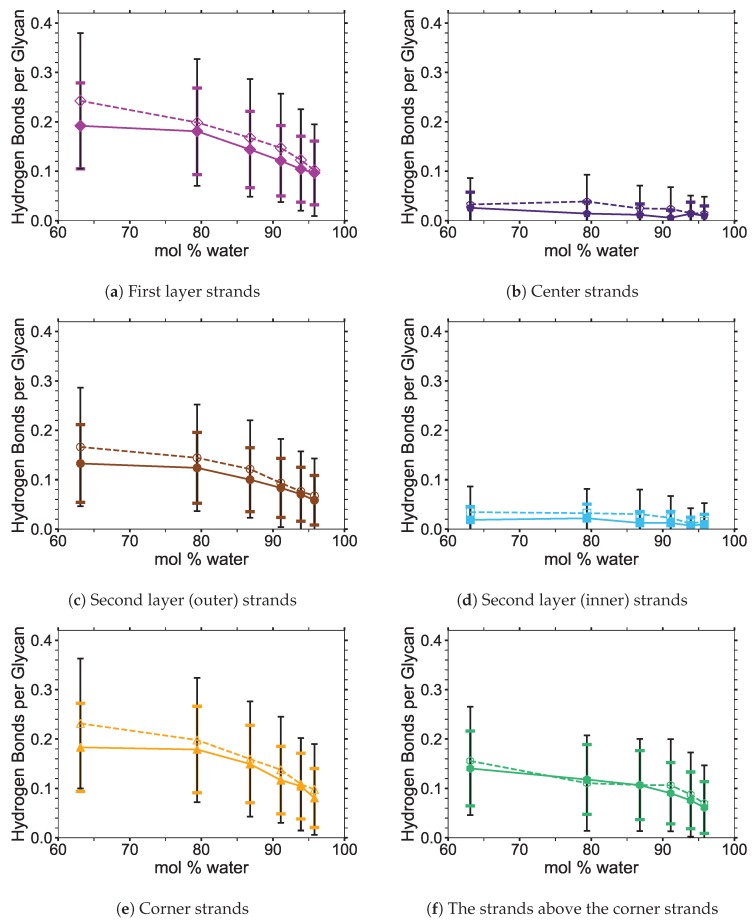
Average **TBP-strand** hydrogen bonding comparison with standard deviations between the small and large cellulose bundles at various water concentrations: (**a**) the first layer strands; (**b**) the center strands; (**c**) the second layer (outer) strands; (**d**) the second layer (inner) strands; (**e**) the corner strands; (**f**) the strands above the corner strands. All data points were averaged from 10 to 20 ns. The solid lines, filled markers, and colored error bars represent the large cellulose bundle, and the dashed lines, unfilled marker, and black error bars show the small cellulose bundle. The colored strands are matched and compared between the plots for the small and large bundles and the images to identify similar hydrogen bonding behavior based on their locations (see Figure 6a,b for the color-coded images). The black cellulose strands are not compared.

**Figure A5:**
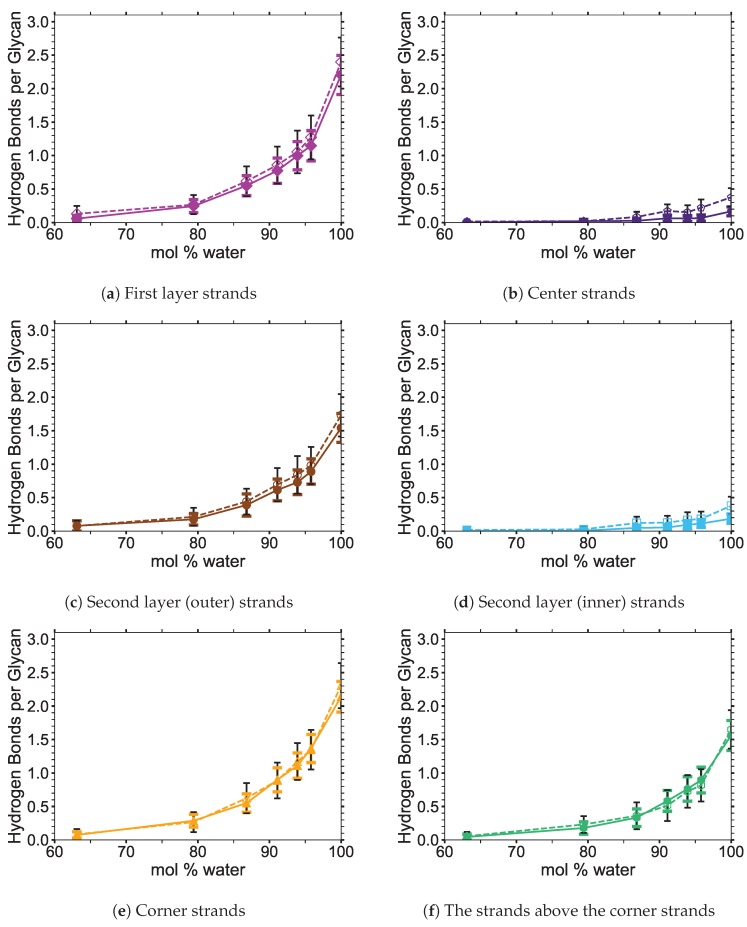
Average **water-strand** hydrogen bonding comparison with standard deviations between the small and large cellulose bundles at various water concentrations: (**a**) the first layer strands; (**b**) the center strands; (**c**) the second layer (outer) strands; (**d**) the second layer (inner) strands; (**e**) the corner strands; (**f**) the strands above the corner strands. All data points were averaged from 10 to 20 ns. The solid lines, filled markers, and colored error bars represent the large cellulose bundle, and the dashed lines, unfilled marker, and black error bars show the small cellulose bundle. The colored strands are matched and compared between the plots for the small and large bundles and the images to identify similar hydrogen bonding behavior based on their locations (see Figure 6a,b for the color-coded images). The black cellulose strands are not compared.

The stability of the cellulose bundle is dependent on the intra-strand (within a strand) and inter-strand (between strands) hydrogen bonding network [3,4,60]. Recent studies have shown that breaking the intra-strand hydrogen bonds is the critical step in the cellulose dissolution process [3,4,60]. Rabideau et al. showed that while the inter-strand hydrogen bonds are quickly broken in a cellulose strand, the cellulose strand does not peel away from the bundle until the intra-strand hydrogen bonds are broken [4]. Therefore, breaking the intra-strand hydrogen bonds appears to be the upper threshold to cellulose dissolution [4]. In this study, the critical intra-strand hydrogen bonding was the same between the solvent-exposed layers (i.e., outer layers or the first and second layers, corner strands, and the strands above the corner strands) of the small and large bundles, while there were significant differences for the inter-strand hydrogen bonding (see Figure 6 and Figure A1 and Figure A2). This difference in the inter-strand hydrogen bonding could be due to the additional twisting of the small cellulose bundle, as shown by Hadden et al. using the GLYCAM06 force field [19]. Hadden et al. also showed that the number of strands in a cellulose bundle stabilizes it from twisting more than the number of glycans per strand, at least for strands with 20 glycans or more in a water solution [19]. Estimating the twisting in these simulations with the data from Hadden et al. yields an approximated 6 % and 1 % twisting of the small and large bundles, respectively. The experimental cellulose twisting provided by Hanley et al. was approximately 0.25 % for a 720-strand bundle [19,20]. Rabideau et al. showed the intra-strand hydrogen bonding is critical to cellulose dissolution using a much smaller cellulose bundle (a 10-strand Iβ bundle with 8 glycans per strand) [4]. This study also compares the TBPCl–water (solvent) hydrogen bonding between the small and large cellulose bundles, which show the same hydrogen bonding between the small and large cellulose bundles (see Figure 7 and Figure A3, Figure A4, Figure A5). The small cellulose bundle size was increased from that of past simulations, which used ILs to study cellulose dissolution [4]. Since the large bundle simulations are computationally infeasible for the long simulations, and the small bundle simulations have the same intra-strand and solvent hydrogen bonding for the solvent-exposed cellulose strands, this justifies the 6-fold reduction in the computation cost of the simulations.

Figure 6 shows the averaged intra-strand and inter-strand hydrogen bonds per glycan, which normalized the data between the small and large bundles for the comparison across a range of water concentrations. The data were averaged from 10 to 20 ns, since only the center strands in the small bundle were not stabilized after 10 ns. The inter-strand hydrogen bonding of the center strands was stabilized in both the small and large bundles within 10 ns. The intra-strand hydrogen bonding of the center strands within the large bundle was stabilized within 3 ns, but the small bundles center strands did not stabilize even after 20 ns. Therefore, the intra-strand hydrogen bonding of the center strands in the small cellulose bundle should stabilize at a lower value than listed in Figure 6d and Figure A1b.

The intra-strand hydrogen bonding was approximately the same between the small and large bundles at all the concentrations, except for the center strands (see Figure 6a–d and Figure A1). The solvent-exposed strands had approximately the same intra-strand hydrogen bonding between the small and large bundles, due to the lack of stabilization from hydrogen bonding between neighboring strands (i.e., the absence of neighboring strands on the solvent-exposed side). The center strands of the large bundles are stabilized better by their surrounding strands since the overall number of hydrogen bonds is higher, and the strands are further away from the solvent. Both of these items increase the structural support of the large bundle with increasing bundle thickness. At the same time, the large bundle also does not appear to be as twisted as the small bundle. The intra-strand hydrogen bonding of the center strands is starkly different between the small and large cellulose bundles, with the large bundle retaining nearly 85 % of its hydrogen bonds (approximately two per glycan is perfect) and coming to equilibrium in 3 ns, while the small bundle has not come to equilibrium and only retained approximately 60 % of its hydrogen bonds at 20 ns. Overall, the cellulose bundle’s first or second layers that dissolve in the simulations will have the same intra-strand hydrogen bonding for both the small and large cellulose bundles.

The inter-strand hydrogen bonding is significantly different between the small and large cellulose bundles, except at 63.1 mol % water (see Figure 6a,b,e,f, and Figure A2). However, at 63.1 mol % water the inter-strand hydrogen bonds are drastic outliers from the rest of the concentrations, with all the values between the small and large bundles being nearly identical. These outliers suggest that lower water concentrations may produce very similar results between the small and large cellulose bundles. In this work, the difference in the cellulose twisting was not quantified between the differing TBPCl–water concentrations. However, the small cellulose bundle does visually appear more twisted than the large cellulose bundle for all the concentrations (see Figure 6a,b). In agreement with these data, the cellulose structure twisting is well documented for the GLYCAM06 force field in pure water and under vacuum [31], being more pronounced for small cellulose bundles and under vacuum conditions [19,20,21].

The number of hydrogen bonds between the TBPCl–water solvent and the cellulose bundle is also an important comparison point. The hydrogen bonds were compared between the Cl, TBP, and water to the strands for both the small and large cellulose bundles. This comparison shows that the solvent-strand hydrogen bonding for the small and large cellulose bundles was nearly the same (see Figure 7). Additionally, the standard deviations for the solvent-strand hydrogen bonding can be found in Figure A3, Figure A4, Figure A5. Since the first layer and corner strands are more solvent-exposed and have the most solvent-strand hydrogen bonds (i.e., interaction with the solvent), they should have the highest probability of strand separation and dissolution.

Using a small cellulose bundle, the GLYCAM06 force field may be capable of identically simulating the outer strand hydrogen bonds of a full-scale cellulose bundle for low water concentration ILs [31]. However, the replication of identical simulations is likely dependent on the attractive Van der Waals (VDW) interactions of the individual IL–water solution, as pointed out by Hadden et al. in a pure water solution [19]. Alternatively, this could mean that even larger cellulose bundles are required to simulate some low water concentration ILs, or this 63.1 mol % water with the large cellulose bundle simulation is a statistical outlier. It is also important to point out that the vacuum space surrounding the cellulose (i.e., the void space or absence of very close molecules around the cellulose due to the deletion of the solvent in the area) increased at the start of these simulations, as the water concentration decreased. This vacuum space increase was unavoidable due to more TBP molecules in the solution and deleting the solvent to insert the cellulose. Also, due to the shape of TBP, the molecular void spaces between the TBP arms increase with decreasing water concentrations, creating potential vacuum spaces or attractive VDW interaction disruptions [12]. Further study of the GLYCAM06 force field is required to confirm that the smaller and larger cellulose bundles outer strands produce similar results for ILs at low water concentrations [31].

This study suggests that the GLYCAM06 force field is capable of adequately simulating the solvent-exposed layers of a large cellulose bundle by only using a small cellulose bundle, at least in the TBPCl–water solution at these water concentrations [21,31]. The data show that the intra-strand hydrogen bonding was the same between the solvent-exposed cellulose strands, while the inter-strand hydrogen bonding was different between the small and large cellulose bundles. Specifically, the small bundles are a reasonable substitute for the large bundles, because the breaking of the intra-strand hydrogen bonds is the critical step in the cellulose dissolution process [3,4,60]. The TBPCl–water (solvent)–strand hydrogen bonding between the small and large cellulose bundles also produced nearly identical results. These results may also apply to other IL-water or IL co-solvent combinations, but more research is required to make a definitive conclusion. It is currently not computationally feasible to simulate the large cellulose bundles in the TBPCl–water solution at various concentrations beyond 100 ns, let alone 600 ns. For the TBPCl–water concentrations studied in this paper, most of the critical system dynamics occur after 100 ns, so simulating the large cellulose bundles even up to 100 ns would provide minimally useful data. Since all the hydrogen bonding between the small and large cellulose bundles is nearly identical, except for the center strands and the inter-strand hydrogen bonding, this is enough to justify the six-fold reduction in the computational cost, which make the simulations computationally possible. A continued hydrogen bonding evolution throughout these simulations is expected, as the GLYCAM06 force field is slowly changing over time and never fully stabilizing in a water solution even after 800 ns or longer [21,31].

## 4. Results: Long Simulations with the Small Iβ Cellulose Bundle

Despite the slow cellulose dissolution due to the moderate temperature, critical data could be obtained about the cellulose dissolution process. The limited cellulose breakdown is primarily attributed to the lower temperature simulations and the computational limits of the system size. The radius of gyration was calculated and was essentially constant, which is accredited to only a few cellulose strands partially dissolving, and the larger size of the bundle.

The key findings of this study are summarized below and explained in more detail in their respective sections. An estimated cellulose dissolution profile was generated for the TBPCl–water solution, in which the cellulose solubility appears to decrease with added water. This study determined that critical hydrogen bonding lifetime values must be maintained between the chloride and the cellulose’s hydroxyl groups that are responsible for its intra-strand hydrogen bonding, in order for cellulose dissolution to occur. The hydrogen bonding lifetime threshold between the chloride–cellulose hydroxyl groups appear to be directly related to cellulose dissolution. The simulations calculated the TBP-cellulose strand pairwise energy, which is favorable (negative) and potentially net negative during the entire cellulose dissolution process. The TBP-cellulose strand pairwise energy shows a favorable thermodynamic pathway for cellulose dissolution in the TBPCl–water solution. Lastly, a cooperative cellulose dissolution mechanism is visualized and determined from these simulations, in which water appears to assist in the cellulose dissolution.

### 4.1. The Extent of Cellulose Dissolution

Each cellulose strand contains 12 glycans (12 glucose units), and they are numbered in a linear order from 1 to 12, in the same direction for every strand. The centers of mass (COMs) of every glycan unit were calculated. For each strand, the distance between the COMs was measured for each matching glycan number, saving the minimum value or nearest neighbor distance between each of the 18 strands (i.e., 17 values per glycan number per strand). For a given glycan number, the maximum nearest neighbor distance is simply the largest of the nearest neighbor distance for all the strands (i.e., the largest nearest neighbor value for the 18 strands). In other words, the maximum nearest neighbor distance is the maximum distance between each nearest COM with a matching glycan number. The average nearest neighbor distance is the nearest neighbor length for each similarly numbered glycan averaged over the different cellulose strands. Only the maximum COM separation distances of the glycan’s nearest neighbor are displayed instead of the average COM distances, as averaging the data at this stage of the dilution process statistically minimizes any useful results.

#### 4.1.1. Cellulose Dissolution

In this work, the amount of dissolved cellulose was calculated using the pure water simulation at 10, 200, 400, and 600 ns as a mathematical basis to determine if each glycan is dissolved in the solution. At these time points, the overall average of the maximum nearest neighbor distances was 6.51 Å and the maxima of all the values were 6.96 Å, with a standard deviation of 0.16 Å. The total glycan COM separation distance determining cellulose dissolution was 7.16 Å, four standard deviations from the average, providing a statistically significant separation.

**Figure 8:**
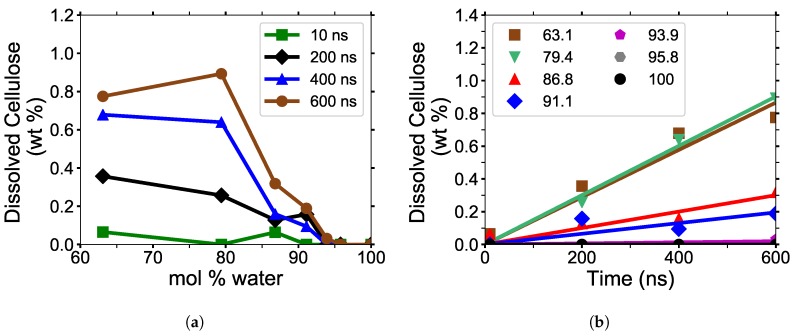
The simulated cellulose dissolution concentrations for the TBPCl–water solution at 360 K: (**a**) cellulose dissolution vs. water concentration; (**b**) cellulose dissolution vs. time (dissolution rates). The legend in plot b indicates the water concentration in mol % water for the TBPCl–water solutions, while the slopes convey the cellulose dissolution rate.

**Figure A6:**
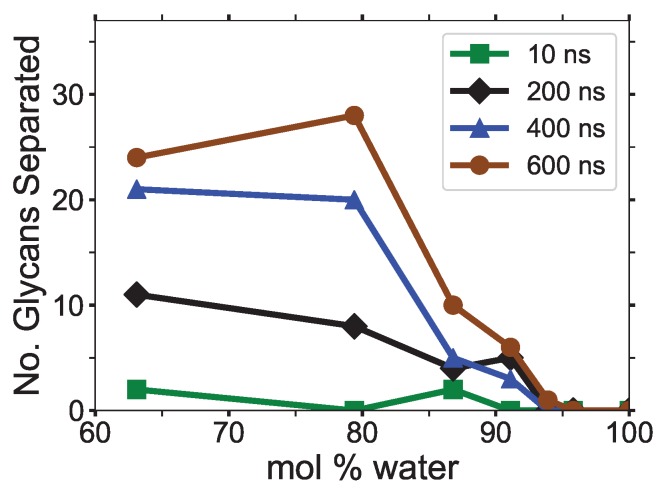
TBPCl–water small cellulose bundles dissolution measured by the number of dissolved glycans at 360 K.

The simulation data appear to be more representative of the TBPCl-DMF experimental data [3], and not statistically maximizing at any specific concentration (see Figure 1b, Figure 8 and Figure A6). The TBPCl–water simulation shows a rapidly decreasing cellulose solubility after 79.4 mol % water. Figure 8b shows a very linear cellulose dissolution rate at the simulated time scales. The dissolution rates appear clustered, according to their ability to dissolve cellulose, grouped with the low water concentrations, (63.1 to 79.4 mol % water), the middle water concentrations (86.8 to 91.1 mol % water), and the high water concentrations (93.9 to 100 mol % water). An anomalous to near-normal diffusion regime transition appears to profoundly impact the middle group where the water veins form throughout the solution, from roughly 80 to 92.5 mol % water [12]. The diffusion regime change with the formation of water veins increases the diffusion of the solvent by approximately an order of magnitude, which could partially explain the cellulose solubility extension into the water concentrations above 79.4 mol % water [12], even with water decreasing the cellulose solubility. The experimental cellulose dissolution profile is estimated to look like Figure 8a based on these simulations and the experimental data from Figure 1b [3]. However, the actual experimental cellulose dissolution profile could be different, as these simulations are far from reaching equilibrium.

#### 4.1.2. Cellulose Strand Separation

**Figure 9:**
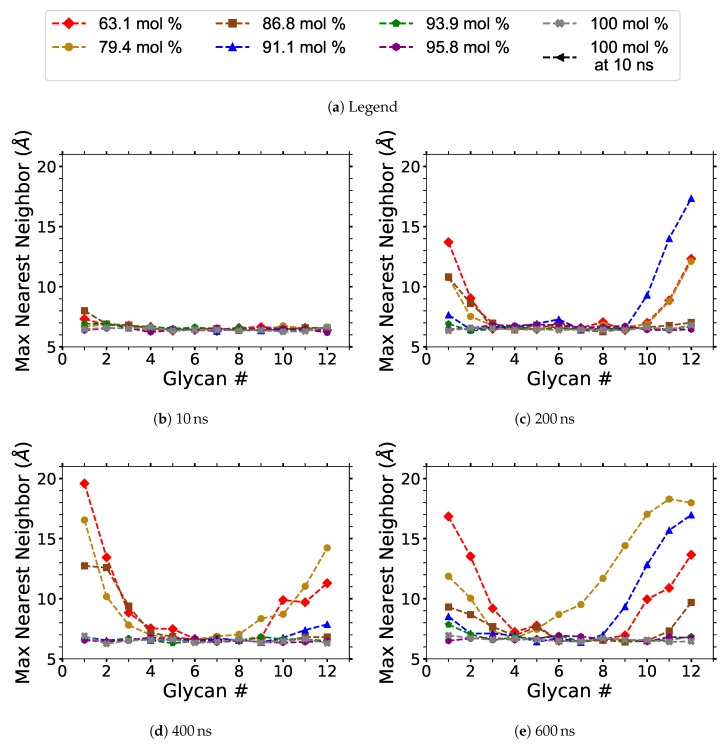
Maximum cellulose strand separation of the small cellulose bundles: (**a**) legend; (**b**) strand separation at 10 ns; (**c**) strand separation at 200 ns; (**d**) strand separation at 400 ns; (**e**) strand separation at 600 ns.

**Figure A7:**
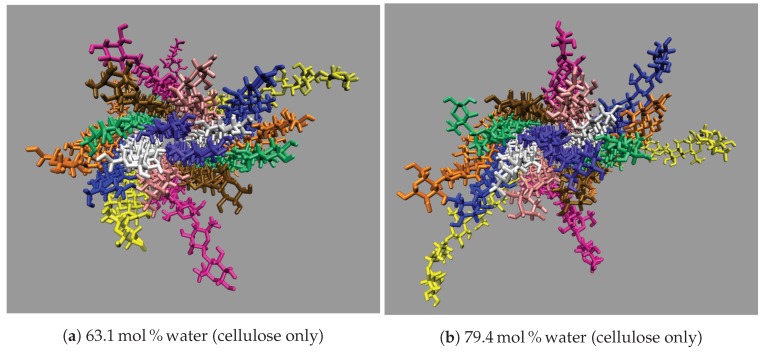

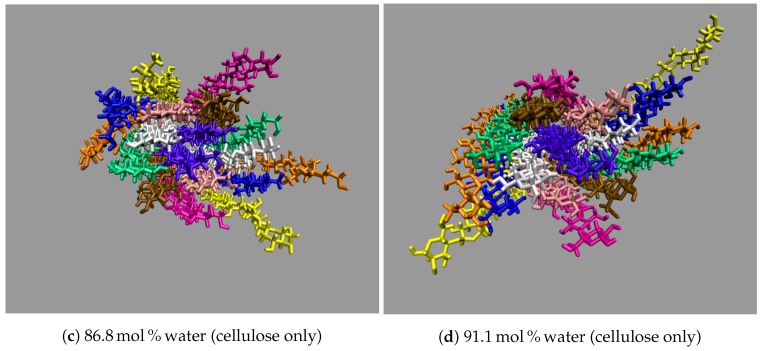
End view images of the cellulose bundle dissolution at 600 ns, showing only the cellulose bundle (Part 1 of 2) at: (**a**) 63.1 mol % water; (**b**) 79.4 mol % water; (**c**) 86.8 mol % water; and (**d**) 91.1 mol % water.

**Figure A8:**
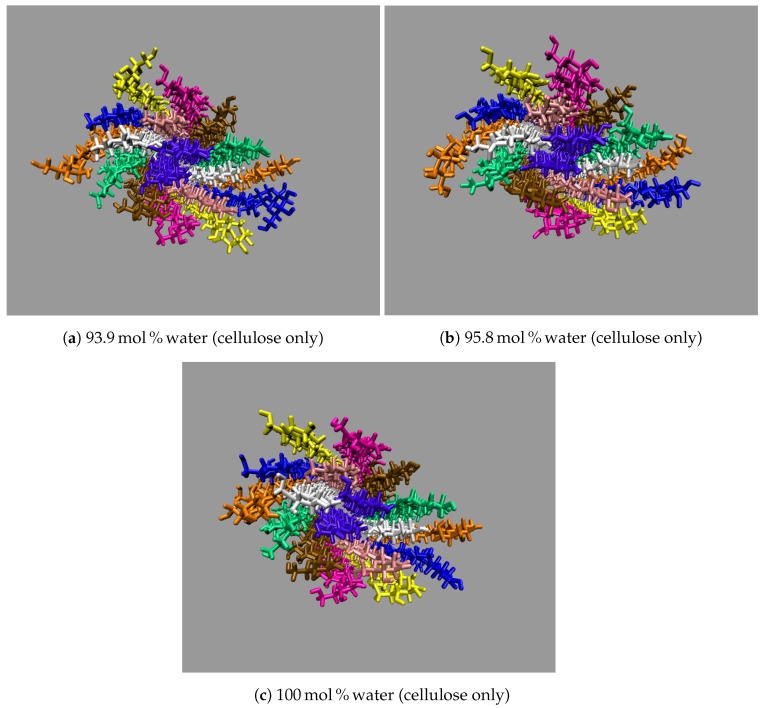
End view images of the cellulose bundle dissolution at 600 ns, showing only the cellulose bundle (Part 2 of 2) at: (**a**) 93.9 mol % water; (**b**) 95.8 mol % water; and (**c**) 100 mol % water.

**Figure A9:**
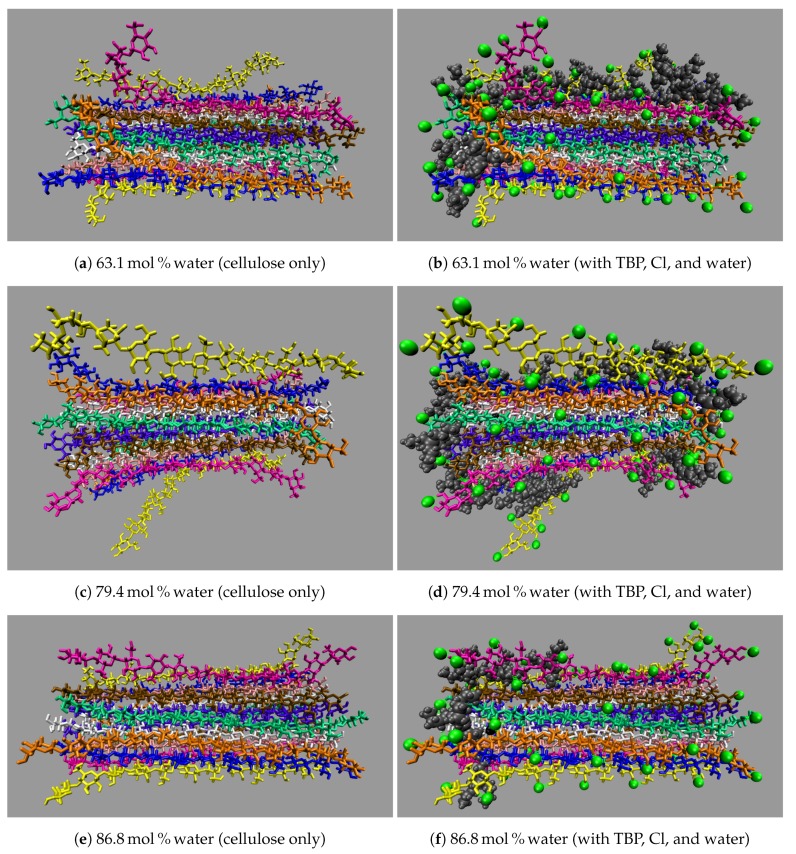
Side view images of the cellulose bundle dissolution at 600 ns, showing all the nearby TBP, Cl, and water molecules as described earlier (Part 1 of 2) at: (**a**) 63.1 mol % water (cellulose only); (**b**) 63.1 mol % water (with TBP, Cl, and water); (**c**) 79.4 mol % water (cellulose only); (**d**) 79.4 mol % water (with TBP, Cl, and water); (**e**) 86.8 mol % water (cellulose only); and (**f**) 86.8 mol % water (with TBP, Cl, and water).

**Figure A10:**
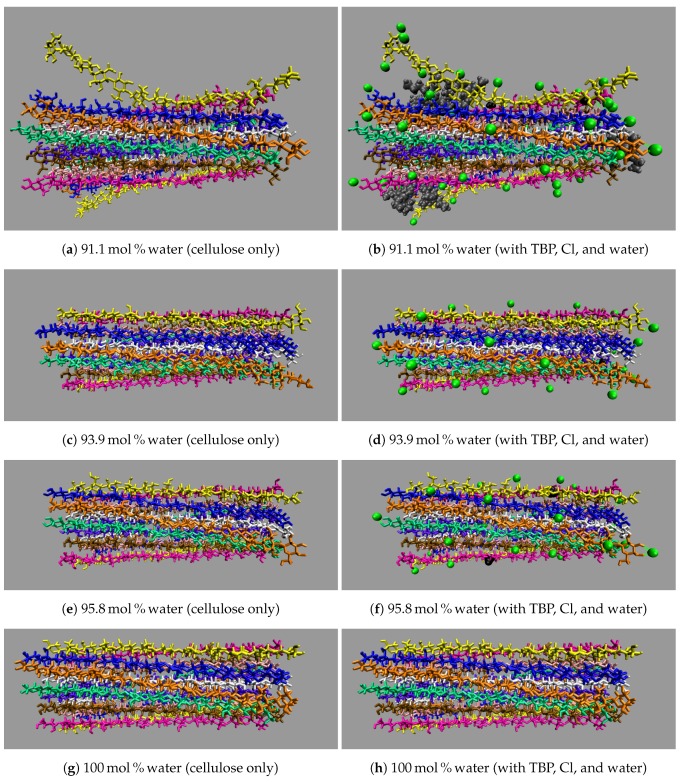
Side view images of the cellulose bundle dissolution at 600 ns, showing all the nearby TBP, Cl, and water molecules (Part 2 of 2) at: (**a**) 91.1 mol % water (cellulose only); (**b**) 91.1 mol % water (with TBP, Cl, and water); (**c**) 93.9 mol%water (cellulose only); (**d**) 93.9 mol % water (with TBP, Cl, and water); (**e**) 95.8 mol % water (cellulose only); (**f**) 95.8 mol % water (with TBP, Cl, and water); (**g**) 100 mol % water (cellulose only); and (**h**) 100 mol % water (with TBP, Cl, and water)

**Figure A11:**
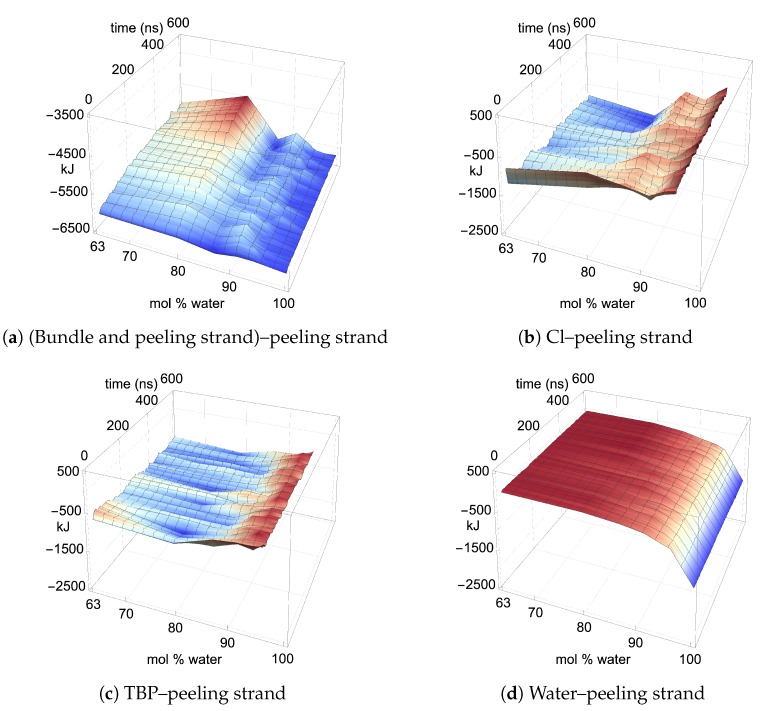
The pairwise energies for the second yellow separating strand at 360K. The pairwise energies are shown between the following: (**a**) (bundle–peeling strand and peeling strand)–peeling strand (i.e., within the strand and with the rest of the cellulose bundle); (**b**) Cl–peeling strand; (**c**) TBP–peeling strand; (**d**) water–peeling strand. These data represent the second yellow strand to peel or the non-peeling yellow strand if the second yellow strand does not peel in the simulation. The data were averaged over 1000 data points, using a rolling average. Every 100th point was then plotted to maintain plot clarity [64].

The maximum nearest neighbor distances between matching glycan numbers are an indicator of a cellulose strand separation from the cellulose bundle and hence cellulose dissolution (see Figure 9). The most probable strands to separate are the yellow and pink strands (i.e., first layer strands), with the yellow strands separating first in the majority of the simulations (see Figure A7, Figure A8, Figure A9, Figure A10, Figure A11). The most probable strands to separate also have the lowest intra-strand and inter-strand hydrogen bonding and the highest hydrogen bonding with the solvent, which are the first layer strands and the corner strands (see Figure 6 and Figure 7).

During the dissolution process, strands can separate and partially or entirely reform. The more significant the cellulose strand separation, the higher the chance the strand will only partially reform, increasing the probability of further separation. This partial strand reformation is seen at 91.1 mol % water between 200 to 400 ns, at both ends of the glycan numbers. In general, the cellulose strands proceed to dissolve, despite some strand reformation. The ends are more likely to peel off than the center of the cellulose bundles, which is an expected result since the ends have fewer hydrogen bonds stabilizing them. The 63.1 and 79.4 mol % water simulations do not always show the furthest strand separation, despite having the highest concentration of dissolution, which means that they have more separating strands than the 91.1 mol % water solution.

### 4.2. Hydrogen Bonding Lifetimes

**Figure 10:**
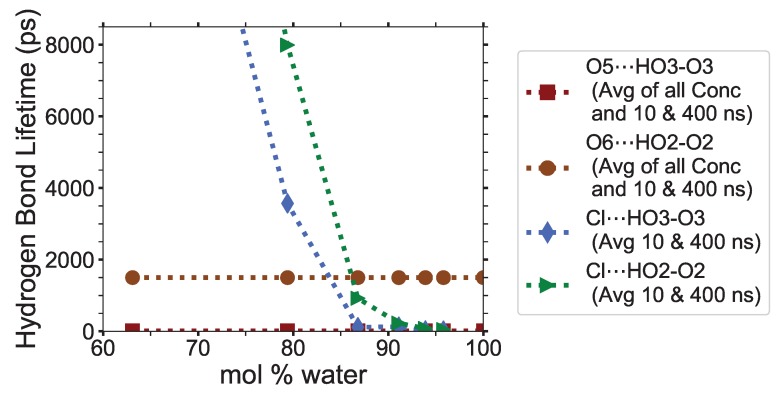
The chloride’s intra-strand hydrogen bond breaking lifetimes vs. the cellulose’s intra-strand hydrogen bonding lifetimes. The cellulose’s intra-strand hydrogen bonding, O5⋯HO3-O3 and O6⋯HO2-O2, should remain relatively constant as it is mostly undissolved. Therefore, the intra-strand hydrogen bonding were averaged for all the concentrations and sample times (10 and 400 ns). The chloride–cellulose hydroxyl oxygen’s hydrogen bonds, Cl⋯HO3-O3 and Cl⋯HO2-O2, were averaged for the 10 and 400 ns times to yield less time-correlated data.

This work refers to the hydrogen bond lifetimes in the following notation: A⋯H-D (hydrogen Acceptor atom⋯Hydrogen atom–hydrogen Donor atom). The hydrogen and hydrogen donor, H-D, are in the same molecule and share a covalent bond. The corresponding A⋯H-D atomic labeling is provided in Figure 2 and Figure 3. For example, the atoms in the TBP molecules are as follows: CTs are the carbons; HP and HCs are the hydrogens; P is the phosphorus. The most critical hydrogen bonding lifetimes are those between the chloride–cellulose hydroxyl hydrogens (Cl⋯HO2-O2 and Cl⋯HO3-O3). When the chloride–cellulose hydroxyl hydrogen lifetimes fall below the cellulose’s largest intra-strand hydrogen bonding lifetime (O5⋯HO3-O3 and O6⋯HO2-O2), cellulose dissolution rapidly declines (see Figure 10).

The hydrogen bonding lifetimes were calculated between the Cl–bundle, TBP–bundle, water–bundle, bundle–bundle, Cl–CT, Cl–water, and CT–water pairs (see Table 3 and Table 4 and Table A2, Table A3, Table A4). The data contain high variations due to the limited time snapshots from which the lifetimes were obtained. Therefore, it is essential to look at the average values and trends and not at single points. Not every possible combination of hydrogen bonding lifetimes was analyzed, so the analysis and comparisons are solely based on the analyzed hydrogen bonding lifetimes, which for the study were determined to be the most significant. The significance was qualitatively determined by analyzing most of the probable combinations and removing the potential hydrogen bonding lifetimes that would be very short. This qualitative method estimated the hydrogen bonds based on their atomic charges but is prone to human errors, so there could be other hydrogen bonding lifetimes that play a role in the dissolution of cellulose in the TBPCl–water solution.

The anion-cellulose carbons (Cl⋯H’s-C’s) have relatively average hydrogen bonding lifetimes across all concentrations (0.490 to 1.13 ps), as seen in Table 3. The anions-cellulose hydroxyl oxygen pairs, Cl⋯HOx’s-Ox’s, have the largest solvent–cellulose hydrogen bonding lifetime, and are listed in order of decreasing strength: Cl⋯HO2-O2, Cl⋯HO3-O3, and Cl⋯HO6-O6, for which all decay in strength with increasing water concentrations. The hydrogen bonding lifetimes across all concentrations for the Cl⋯HO2-O2, Cl⋯HO3-O3, and Cl⋯HO6-O6 pairs last for 48.9 to > 31445 ps, 8.20 to > 20848 ps, and 11.3 to 7189 ps, respectively. These Cl⋯HOx’s-Ox’s hydrogen bonds last many orders of magnitude longer than any other hydrogen bonding pairs, except the inter cellulose hydrogen bond between O4⋯H2-C2 (see Table A3), allowing the chloride to greatly interrupt the cellulose intra-strand hydrogen bonding between the O5⋯HO3-O3 and O6⋯HO2-O2 pairs [3]. The O5⋯HO3-O3 and O6⋯HO2-O2 hydrogen bonding lifetimes are 2.88 to 18.8 ps and 62.6 to 3318 ps, respectively (see Table 4).

The Cl⋯HO2-O2 and Cl⋯HO3-O3 have greater hydrogen bonding lifetimes than the intra-strand hydrogen bonding pairs (O5⋯HO3-O3 and O6⋯HO2-O2) between 63.1 to 79.4 mol % water. However, they start to move below intra-strand lifetimes between 79.4 to 86.8 mol % water, where the cellulose dissolution power of the solution begins to weaken dramatically (see Figure 10). Once the Cl⋯HO2-O2 and Cl⋯HO3-O3 are very weak and fairly stable, around 12 to 104 ps, the solution is no longer capable of dissolving cellulose. The anion⋯HO2-O2 and anion⋯HO3-O3 lifetimes, or similar intra-strand hydrogen bond disrupters, could be a parameter programmed into the simulations to calculate where it falls below the intra-strand bonding lifetimes, thereby indicating where the cellulose dissolution power of the IL co-solvent may begin to decline and vanish.

The Cl⋯HOx’s-Ox’s hydrogen bond’s high strength helps in disrupting the intra-strand and inter-strand hydrogen bonds [4]. The bonding of the cellulose strands to chloride, or similar anions, appears to loosen them from the bundle and assist in their dissolution [3,4,60]. The most drastic disruption of all the hydrogen bond lifetimes with increasing water concentrations occurs for the Cl⋯HOx’s-Ox’s pairs (see Figure 10). These hydrogen bond lifetimes drop by approximately 1.5 to 2.3 orders of magnitude from 63.1 to 86.8 mol % water, and finally drop another 0.7 to 1.1 orders of magnitude from 86.8 to 93.9 mol % water. The Cl⋯Hw-Ow hydrogen bond lifetimes also decay rapidly from 21.0 to 2.62 ps with the concentrations changing from 63.1 to 86.8 mol % water, as the chloride’s first solvation shell becomes saturated [12]. The rapid decay of the Cl⋯HO2-O2, Cl⋯HO3-O3, Cl⋯HO6-O6, and Cl⋯Hw-Ow hydrogen bonding strength throughout the increasing water concentrations is presumably a large factor in the ability to dissolve cellulose. However, this anionic loosening of cellulose strands is by no means the only contributing factor to the cellulose dissolution.

The TBP molecules have the least number of hydrogen bonds with the peeling cellulose strand, except for the 63.1 mol % water concentration, where there is not much water. The CT⋯HO’s-O’s and Ox’s⋯H’s-CT’s hydrogen bonding lifetimes are rather small in comparison to the Cl⋯HOx’s-Ox’s hydrogen bonding lifetimes (0.076 to 0.236 ps vs. 6.78 to > 31445 ps). The CT⋯H’s-C’s hydrogen bonding lifetimes are approximately equal to the Cl⋯H’s-C’s lifetimes, which shows that there is little hydrogen bonding preference between them (0.285 to 0.427 ps vs. 0.490 to > 1.13 ps). The CT⋯-H’s-C’s hydrogen bonds are only stronger than some of the cellulose–cellulose hydrogen bonds. By comparing the hydrogen bonding, pairwise energies and the simulation snapshots, it appears that TBP does participate in the cellulose strand separation, at least in part by hydrogen bonding. Based on the hydrogen bond lifetimes, the TBP can hydrogen-bond to the cellulose strands for short durations. The Ox’s⋯H’s-CT’s hydrogen bonds are all about the same strength, with shrinking hydrogen bonding lifetimes for increasing water concentrations. Since the Ox’s⋯H’s-CT’s hydrogen bonds are approximately the same strength, the cellulose oxygens all participate equally in hydrogen bonding, provided there are no steric hinderances. The CT⋯Hw-Ow hydrogen bonding lifetimes are very weak (0.026 to 0.038 ps), indicating that long-term hydrogen bonding is less probable.

The water molecules have steady and moderate hydrogen bonding lifetimes with the peeling cellulose strands. Specifically, the Ow⋯HO2-O2 and Ow⋯HO6-O6 hydrogen bonding lifetimes are the highest for water, producing moderate strengths of 1.05 to 11.3 ps and 0.789 to 12.1 ps, respectively. Water’s Ow⋯HO2-O2 and Ow⋯HO6-O6 hydrogen bonding strengths also decrease with increasing water concentration, dropping by approximately 0.5 orders of magnitude from 79.4 to 93.9 mol % water. The water hydrogen bonding strengths are moderate for the Ow⋯HO2-O2 and Ow⋯HO6-O6 pairs, but weak for the Ow⋯HO3-O3. The Ow⋯HO3-O3 hydrogen bonding lifetimes do not appear to decay with increasing water concentration, falling between 0.026 to 2.58 ps, with the variance or range of values occurring from random sampling with no notable trend. The water hydrogen bonding strengths suggest that the water can assist in the cellulose strand separation in the Ow⋯HO2-O2 and Ow⋯HO6-O6 pairs, but the chloride anion is largely required for the Ow⋯HO3-O3 separation. While water interrupts the cellulose’s hydrogen bonding, it lacks the enhanced duration and ability to maintain multiple strong hydrogen bonds with the cellulose, as the anions do. The water and anion bonding strength to the cellulose’s carbons are about the same (Cl⋯H’s-C’s and Ow⋯H’s-C’s), indicating these hydrogen bonding locations are not a factor to the anion’s dissolution power.

The cellulose–cellulose hydrogen bonding lifetimes are also provided in Table 4, and in the Appendix A (see Table A2, Table A3, Table A4). The intra-strand bonding is much stronger for the O6⋯HO2-O2 pair than for the O5⋯HO3-O3 pair. There are many inter-strand hydrogen bonding lifetimes provided in these data, ranging from weak to very strong. Many of the hydrogen bonding lifetimes decay with an increasing co-solvent (not including the cellulose–cellulose hydrogen bonding lifetimes), in agreement with the tetrabutylammonium acetate (TBAAc) dimethyl sulfoxide (DMSO) simulations, which used a central atom in each molecule to determine the molecular contact lifetimes [60]. The critical evaluation for any IL co-solvent solution is determined by the co-solvent type and the ’working concentration range’ where the solution can effectively dissolve cellulose. For the TBPCl–water solution, the ’working range’ for water would ideally be at a higher concentration to absorb some of the water naturally contained in the biomass. Therefore, a balance likely exists in many IL water solutions where the declining anion⋯HOx’s-Ox’s hydrogen bond’s strength must be balanced with a higher water concentration and other properties that affect cellulose dissolution (i.e., increased diffusion).

### 4.3. Pairwise Energies and Hydrogen Bonding of the Peeling Strands

Throughout the dissolution process, the pairwise energies of the peeling cellulose strands identify the favorable (negative), unfavorable (positive), and neutral contributions from the individual molecular types in the solvent and the rest of the cellulose bundle. During the cellulose dissolution process, the pairwise energies show favorable interactions between the peeling strand and both the Cl and TBP molecules. The pairwise energies between the TBP and peeling strand (TBP–peeling strand) are consistently favorable and potentially net negative, which was not seen for the imidazolium-based ILs [4]. Hence, this may be a critical and unique attribute that enables the TBP-based solutions to dissolve cellulose in high water concentrations.

**Figure 11:**
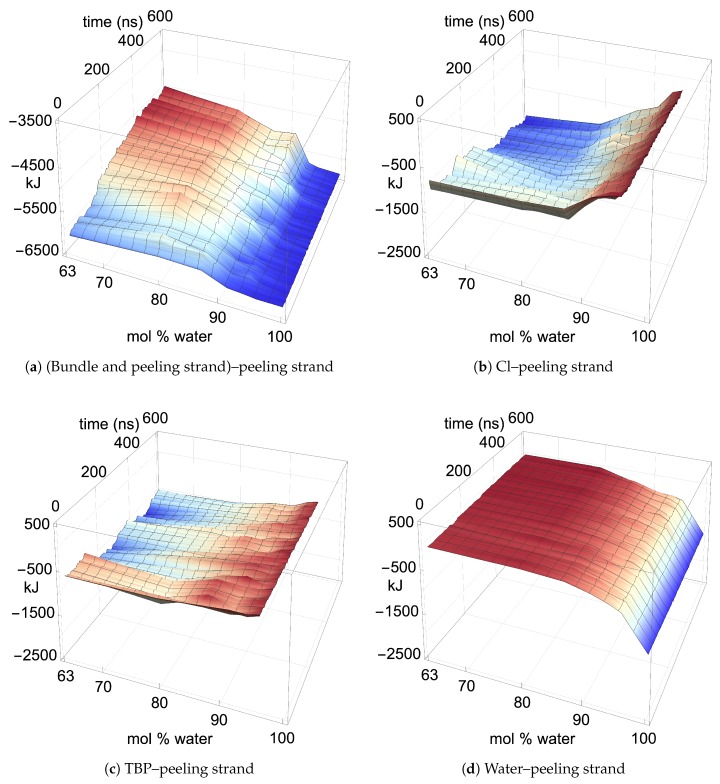
The pairwise energies for the first yellow separating strand at 360 K. The pairwise energies are shown between the following: (**a**) (bundle–peeling strand and peeling strand)–peeling strand (i.e., within the strand and with the rest of the cellulose bundle); (**b**) Cl–peeling strand; (**c**) TBP–peeling strand; (**d**) Water–peeling strand. These data represent the first yellow strand to peel or the non–peeling yellow strand if no yellow strands peeled in the simulation. The data were averaged over 1000 data points, using a rolling average. Every 100th point was then plotted to maintain plot clarity [64].

**Figure 12:**
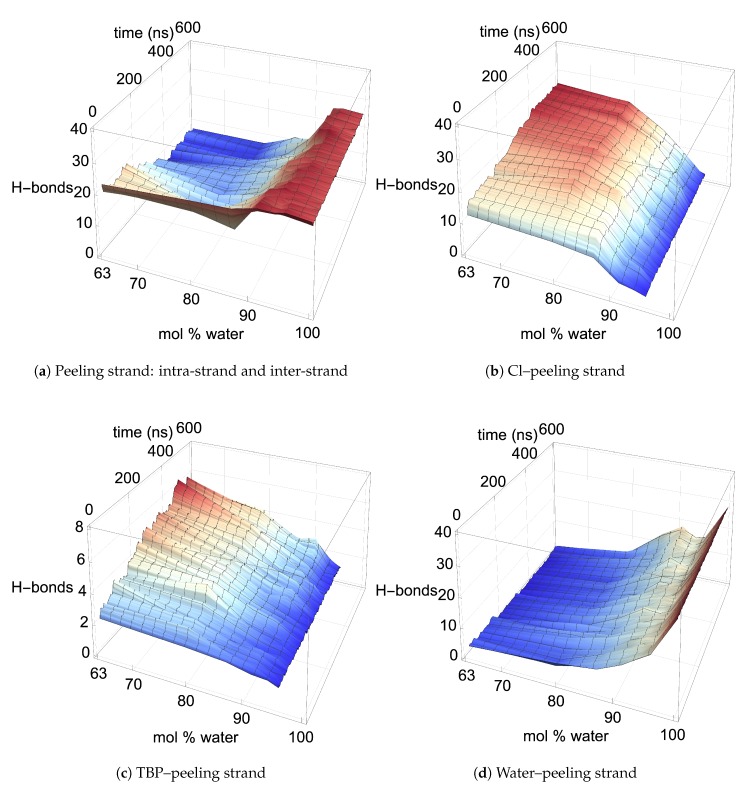
The hydrogen bonding for the first yellow separating strand at 360 K (Part 1 of 2). The hydrogen bonds are shown between the following: (**a**) (bundle–peeling strand and peeling strand)–peeling strand (i.e., intra-strand and inter-strand hydrogen bonds for the separating strand); (**b**) Cl–peeling strand; (**c**) TBP–peeling strand; (**d**) water–peeling strand. These data represent the first yellow strand to peel or the non-peeling yellow strand if no yellow strands peeled in the simulation. The data were averaged over 1000 data points, using a rolling average. Every 100th point was then plotted to maintain plot clarity [64].

**Figure 13:**
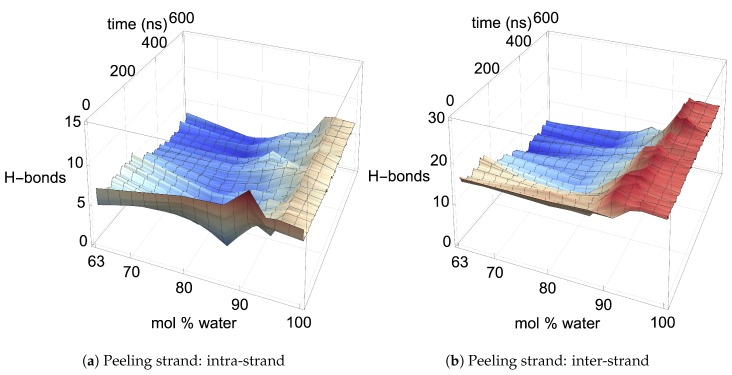
The hydrogen bonding for the first yellow separating strand at 360 K (Part 2 of 2). The hydrogen bonds are shown between the following: (**a**) cellulose: intra–peeling strand (i.e., the intra-strand hydrogen bonds for the separating strand); (**b**) cellulose: inter–peeling strand (i.e., the inter-strand hydrogen bonds for the separating strand). These data represent the first yellow strand to peel or the non-peeling yellow strand if no yellow strands peeled in the simulation. The data were averaged over 1000 data points, using a rolling average. Every 100th point was then plotted to maintain plot clarity. [64].

**Figure A12:**
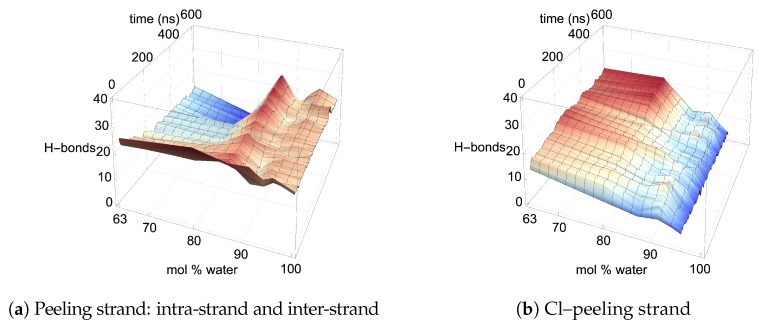

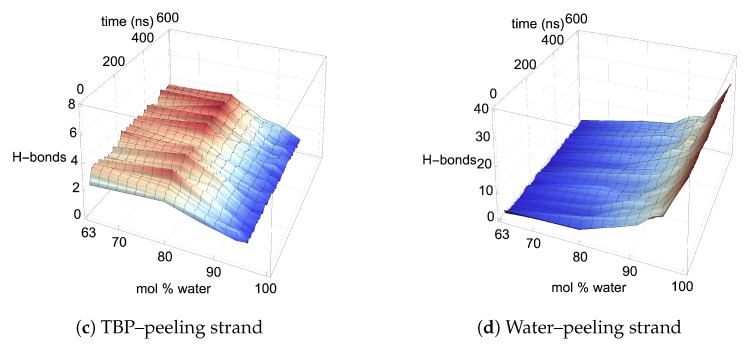
The hydrogen bonding for the second yellow separating strand at 360K (Part 1 of 2). The hydrogen bonds are shown between the following: (**a**) (bundle–peeling strand and peeling strand)–peeling strand (i.e., intra-strand and inter-strand hydrogen bonds for the separating strand); (**b**) Cl–peeling strand; (**c**) TBP–peeling strand; (**d**) water–peeling strand. These data represent the second yellow strand to peel or the non-peeling yellow strand if the second yellow strand does not peel in the simulation. The data were averaged over 1000 data points, using a rolling average. Every 100th point was then plotted to maintain plot clarity [64].

**Figure A13:**
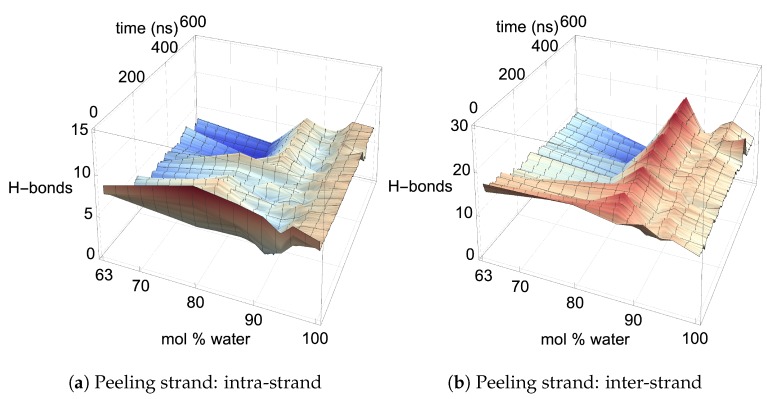
The hydrogen bonding for the second yellow separating strand at 360 K (Part 1 of 2). The hydrogen bonds are shown between the following: (**a**) (bundle–peeling strand and peeling strand)–peeling strand (i.e., intra-strand and inter-strand hydrogen bonds for the separating strand); (**b**) Cl–peeling strand; (**c**) TBP–peeling strand; (**d**) water–peeling strand. These data represent the second yellow strand to peel or the non-peeling yellow strand if the second yellow strand does not peel in the simulation. The data were averaged over 1000 data points, using a rolling average. Every 100th point was then plotted to maintain plot clarity [64].

**Figure A14:**
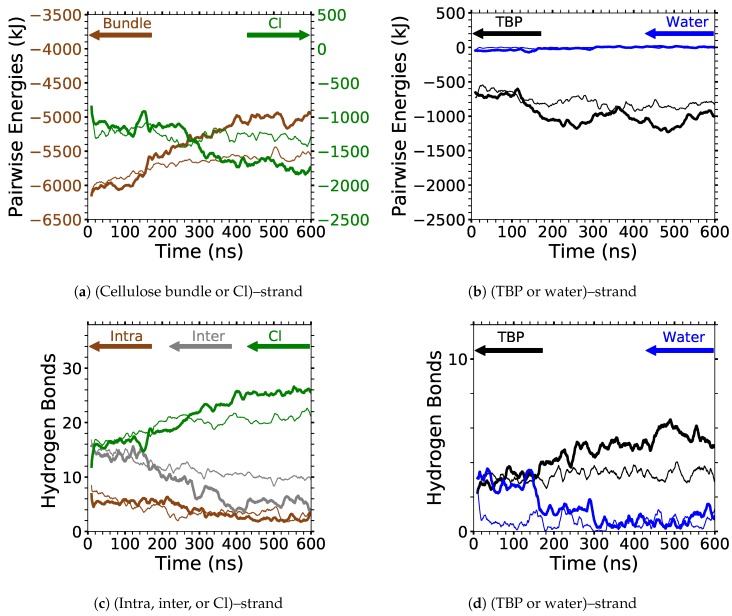
Pairwise energies and hydrogen bonding of the peeling strand(s) at 63.1mol%water and 360K. The pairwise energies of the separating strand(s) between the: (**a**) (cellulose bundle or Cl)–separating strand; (**b**) (TBP or water)–separating strand. The number of hydrogen bonds for the separating strand(s) per the given type: (**c**) (intra, inter, or Cl)–separating strand; (**d**) (TBP or water)–separating strand. The thick line is the first yellow strand to peel, and the thin line is the non-peeling yellow strand or second strand to peel. If neither of the stands peel, then both the thick and thin lines are non-peeling yellow strands. The data were averaged over 1000 data points, using a rolling average.

**Figure A15:**
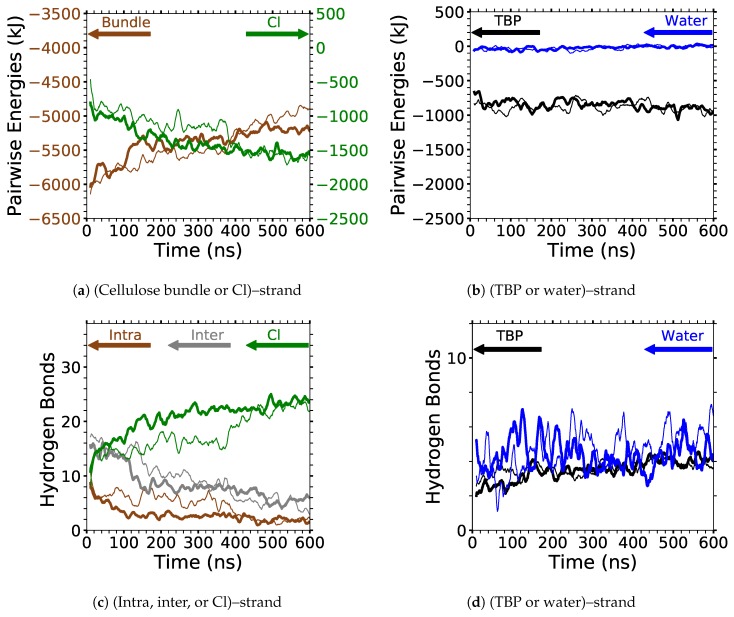
Pairwise energies and hydrogen bonding of the peeling strand(s) at 79.4 mol % water and 360K. The pairwise energies of the separating strand(s) between the: (**a**) (cellulose bundle or Cl)–separating strand; (**b**) (TBP or water)–separating strand. The number of hydrogen bonds for the separating strand(s) per the given type: (**c**) (intra, inter, or Cl)–separating strand; (**d**) (TBP or water)–separating strand. The thick line is the first yellow strand to peel, and the thin line is the non-peeling yellow strand or second strand to peel. If neither of the stands peel, then both the thick and thin lines are non-peeling yellow strands. The data were averaged over 1000 data points, using a rolling average.

**Figure A16:**
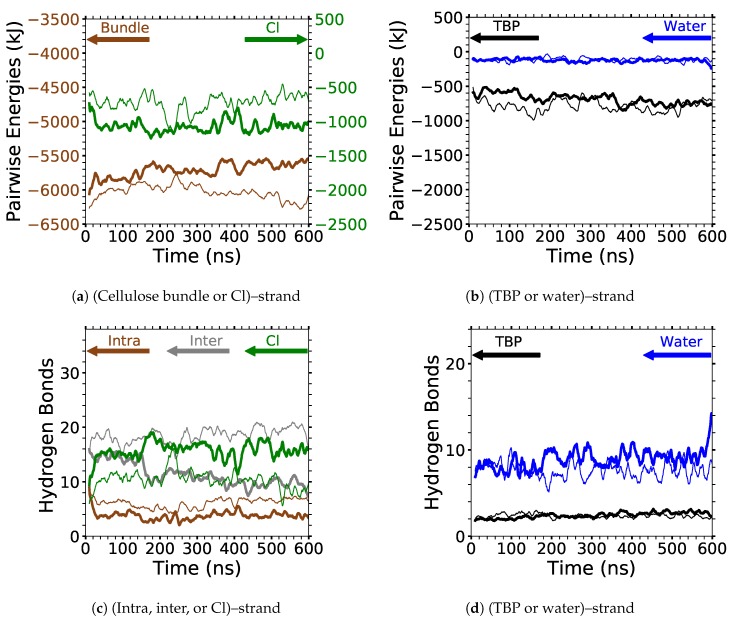
Pairwise energies and hydrogen bonding of the peeling strand(s) at 86.8 mol % water and 360K. The pairwise energies of the separating strand(s) between the: (**a**) (cellulose bundle or Cl)–separating strand; (**b**) (TBP or water)–separating strand. The number of hydrogen bonds for the separating strand(s) per the given type: (**c**) (intra, inter, or Cl)–separating strand; (**d**) (TBP or water)–separating strand. The thick line is the first yellow strand to peel, and the thin line is the non-peeling yellow strand or second strand to peel. If neither of the stands peel, then both the thick and thin lines are non-peeling yellow strands. The data were averaged over 1000 data points, using a rolling average.

**Figure A17:**
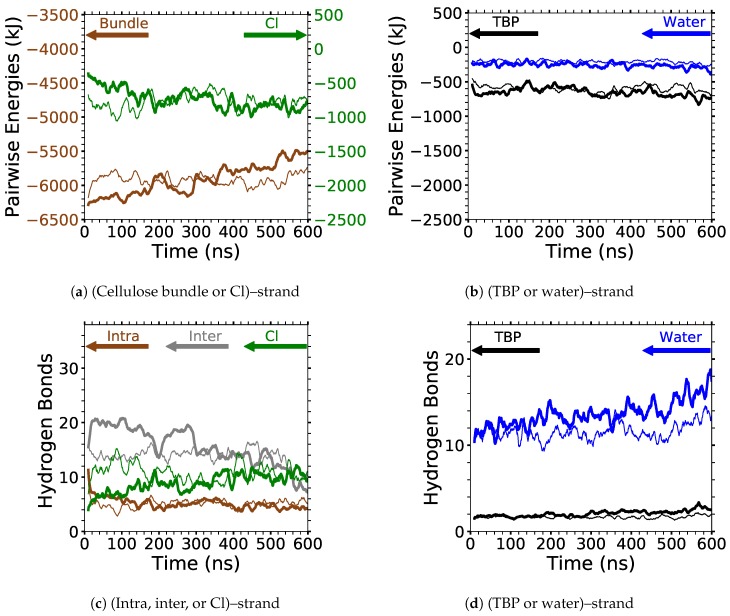
Pairwise energies and hydrogen bonding of the peeling strand(s) at 91.1 mol % water and 360K. The pairwise energies of the separating strand(s) between the: (**a**) (cellulose bundle or Cl)–separating strand; (**b**) (TBP or water)–separating strand. The number of hydrogen bonds for the separating strand(s) per the given type: (**c**) (intra, inter, or Cl)–separating strand; (**d**) (TBP or water)–separating strand. The thick line is the first yellow strand to peel, and the thin line is the non-peeling yellow strand or second strand to peel. If neither of the stands peel, then both the thick and thin lines are non-peeling yellow strands. The data were averaged over 1000 data points, using a rolling average.

**Figure A18:**
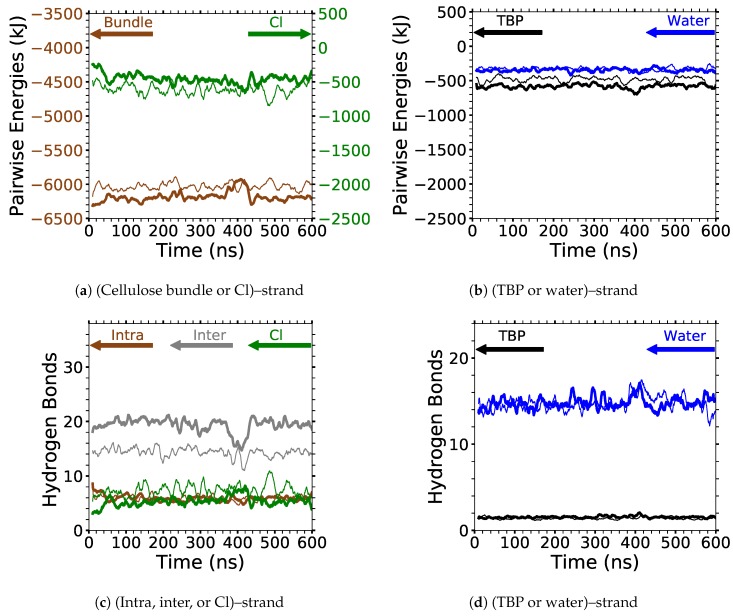
Pairwise energies and hydrogen bonding of the peeling strand(s) at 93.9 mol % water and 360K. The pairwise energies of the separating strand(s) between the: (**a**) (cellulose bundle or Cl)–separating strand; (**b**) (TBP or water)–separating strand. The number of hydrogen bonds for the separating strand(s) per the given type: (**c**) (intra, inter, or Cl)–separating strand; (**d**) (TBP or water)–separating strand. The thick line is the first yellow strand to peel, and the thin line is the non-peeling yellow strand or second strand to peel. If neither of the stands peel, then both the thick and thin lines are non-peeling yellow strands. The data were averaged over 1000 data points, using a rolling average.

**Figure A19:**
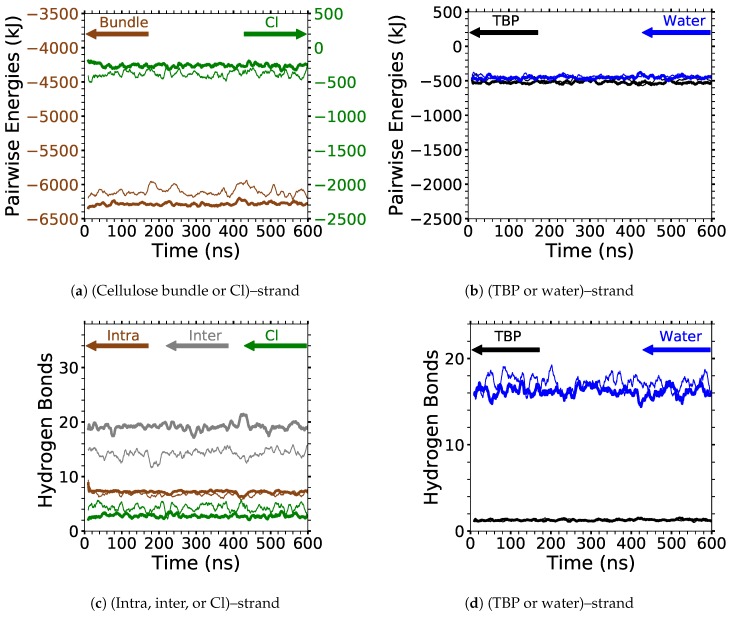
Pairwise energies and hydrogen bonding of the peeling strand(s) at 95.8 mol % water and 360K. The pairwise energies of the separating strand(s) between the: (**a**) (cellulose bundle or Cl)–separating strand; (**b**) (TBP or water)–separating strand. The number of hydrogen bonds for the separating strand(s) per the given type: (**c**) (intra, inter, or Cl)–separating strand; (**d**) (TBP or water)–separating strand. The thick line is the first yellow strand to peel, and the thin line is the non-peeling yellow strand or second strand to peel. If neither of the stands peel, then both the thick and thin lines are non-peeling yellow strands. The data were averaged over 1000 data points, using a rolling average.

**Figure A20:**
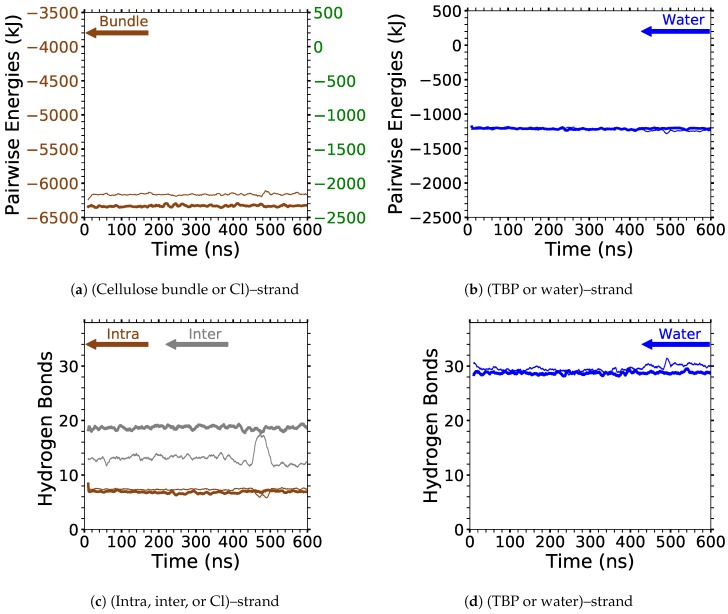
Pairwise energies and hydrogen bonding of the peeling strand(s) at 100 mol % water and 360K. The pairwise energies of the separating strand(s) between the: (**a**) (cellulose bundle or Cl)–separating strand; (**b**) (TBP or water)–separating strand. The number of hydrogen bonds for the separating strand(s) per the given type: (**c**) (intra, inter, or Cl)–separating strand; (**d**) (TBP or water)–separating strand. The thick line is the first yellow strand to peel, and the thin line is the non-peeling yellow strand or second strand to peel. If neither of the stands peel, then both the thick and thin lines are non-peeling yellow strands. The data were averaged over 1000 data points, using a rolling average.

The two yellow strands are used for the pairwise energy and hydrogen bonding comparison as they are the mirror images of one another, and hence, both strands should have the same pairwise energies given that they are at the same dissolution state in the process (see Figure 4). If the cellulose bundle–peeling strand pairwise energies are increasing, then that yellow cellulose strand is peeling. The pairwise energies and hydrogen bonding for the first peeling yellow strand are shown in Figure 11, Figure 12 and Figure 13. The pairwise energies and hydrogen bonding for the second peeling yellow strand are not shown in the main text but can be found in the Appendix A (see Figure A11, Figure A12, Figure A13). Additional two-dimensional pairwise energy and hydrogen bond plots at a fixed concentration are shown in the Appendix A (see Figure A14, Figure A15, Figure A16, Figure A17, Figure A18, Figure A19, Figure A20). The TBP, Cl, and water pairwise energy values are each proportionally attributed to interaction with the peeling cellulose strand.

In the region of dissolution, 63.1 mol % water–91.1 mol % water, the chlorides–peeling strands have a net negative pairwise energy, which offsets the net positive bundle–peeling strand pairwise energy, allowing the cellulose strands to separate via an energetically favorable pathway (see Figure 11a,b and Figure A11a,b) [4]. There is a direct correlation between the net negative chlorides–peeling strand pairwise energies and the formation of chloride–peeling strand hydrogen bonds, indicating that the hydrogen bonding is the primary driver of the net negative pairwise energy (see Figure 11b, Figure 12b, Figure A11b, and Figure A12b) [4]. The bundle–peeling strand net positive pairwise energies are also directly correlated to the loss of hydrogen bonds between the cellulose bundle and the peeling strands [4]. This effect is due to the chloride’s hydrogen bonding to the cellulose strand (see Figure 11a, Figure 12a, Figure A11a, and Figure A12a) [4]. The cellulose strand hydrogen bonding is broken down further between the intra-strand and inter-strand hydrogen bonding in Figure 13 and Figure A13. Both the intra-strand and inter-strand hydrogen bonding are significantly reduced during the strand separation process, where the chloride hydrogen bonds to the peeling strand, unbinding the strand from the cellulose bundle and allowing it to twist as it peels away [4].

Where the TBPCl–water solution has the maximum cellulose dissolution power, the TBP–peeling strand pairwise energies are a favorable and net negative, as shown in Figure 11c and Figure A11c. The large fluctuations in the TBP-separating strand pairwise energies over time, most dramatically seen at 63.1 mol % water, indicate an increasingly negative pairwise energy, followed by a molecular relaxation after the cellulose separates (also see Figure A14). The TBP–peeling strand hydrogen bonds are loosely correlated to the net negative pairwise energies, demonstrating that dispersion or Coulombic forces also drive the net negative pairwise energies and the cellulose separation process (see Figure 11c, Figure 12c, Figure A11c and Figure A12c). The 63.1 mol % water simulation shows a very noticeable net negative pairwise energy during the separation process, while all the other concentrations are approximately neutral to slightly net negative. However, these net negative energies may relax back to the net neutral favorable pairwise energies during longer simulations or with the loss of the hydrogen bonding, similar to other studies [4]. While other studies suggested that the hydrophobic or electrostatic forces of the TBP–peeling strand may allow the cellulose dissolution, this study quantitatively shows the hydrogen bonding, dispersion and Coulombic forces energetically assisting the cellulose dissolution, especially in non-alkali solutions [60,61,62,63].

For much of the I-α and I-β cellulose bundle dissolution in ethyl-3-methylimidazolium acetate (EMIM-Ac), the EMIM–peeling strand has an unfavorable pairwise energy, and the Ac–peeling strand maintains a favorable pairwise energy [4]. For the I-α cellulose bundle, the EMIM–peeling strand and Ac–peeling strand pairwise energies very quickly relax to a net neutral energy contribution after each part of the dissolution, which was not witnessed in this study, although this is a different type of cellulose bundle [4]. In this study, the chloride–peeling strand net negative and the bundle–peeling strand net positive energy interactions are similar to the I-β cellulose bundle dissolution with the EMIM-Ac IL, shown by Rabideau et al. [4]. However, the TBP–peeling strands appear to always have a favorable and increasingly negative pairwise energy, instead of the mostly unfavorable EMIM–peeling strand pairwise energy [4]. Therefore, the slightly net negative and favorable contribution of the TBP–peeling strand pairwise energy could be a unique attribute of the TBPCl–water mixture and the TBP family of ILs.

The water–peeling strand pairwise energy can be slightly net positive, net negative, or net neutral, and is partially determined by the current state of the water–peeling strand hydrogen bonding (see Figure 11d, Figure A11d, and Figure A14, Figure A15, Figure A16, Figure A17, Figure A18, Figure A19, Figure A20). The water–peeling strand pairwise energy can either be favorable or unfavorable at the lower water concentrations, which is dependent on the current state of the cellulose strand separation and the water–peeling strand hydrogen bonding. However, water appears to assist in cellulose dissolution as it can weakly hydrogen-bond to the cellulose strand, providing short-term and slightly lower pairwise energies (see Table 3 and Table 4 for the hydrogen bonding lifetimes). At moderate water concentrations, these water properties may help prevent cellulose strand reformation by allowing water to get between the cellulose strands. Therefore, the approximately net energy neutral and somewhat lower short-term pairwise energies could be a factor in its success in preventing cellulose strand reformation.

### 4.4. Cellulose Dissolution Mechanism

**Figure 14:**
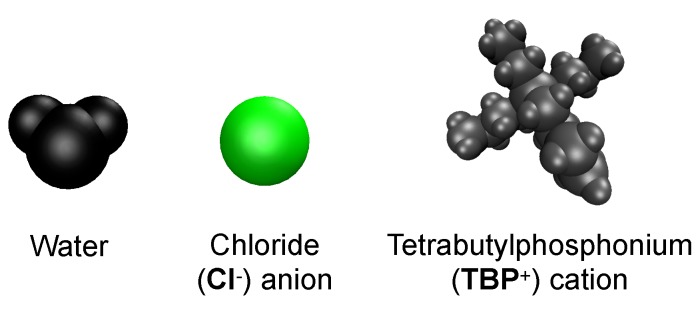
IL and co-solvent molecular representations [23].

All the concentrations that dissolve cellulose reveal the same dissolution mechanism. The first layer strands and the corner strands are the easiest to peel away from the cellulose bundle, as these strands have the least hydrogen bonds stabilizing them (i.e., both intra-strand and inter-strand hydrogen bonds), and they are least sterically hindered from the rest of the cellulose bundle (see Figure 6). Additionally, the first layer strands and the corner strands are the most solvent-exposed, leading to the most probable Cl, TBP, and water interactions (see Figure 7). The images show the greatest cellulose dissolution at 79.4 mol % water, which agrees with the data in Figure 8a,b. As expected, the 100 mol % water simulation shows no cellulose dissolution, but does visually exhibit cellulose bundle twisting like the rest of the concentrations [19,21]. The cellulose dissolution visually appears to be most probable at the ends of the bundles, as the dissolution starts at the ends of the strands, while the center of the strands remains connected (also see Figure 9). The final dissolution images for all the water concentrations at 600 ns can be found in the Appendix A (see Figure A7, Figure A8, Figure A9, Figure A10). The molecular representations of the solvent in the simulation snapshots are provided in Figure 14.

These images selectively depict the IL and co-solvent (solvent) to show the crucial cellulose-solvent interactions (also see Figure 7, Figure 11, Figure 12 and Figure 13 and Figure A11, Figure A12, Figure A13, Figure A14, Figure A15, Figure A16, Figure A17, Figure A18, Figure A19, Figure A20). The interior cellulose strands are the light pink, white, and purple strands in Figure 4. The chloride anions are shown if they are within 3.5 Å of the cellulose bundle. The water and TBP molecules are only shown if they are within 3.5 Å of the 10 inner glycans of the interior cellulose strands. Dashed lines depict hydrogen bonding. The green dashed lines indicate the chloride–cellulose hydrogen bonds and are shown anywhere on the cellulose bundle. The light blue dashed lines indicate the water–cellulose hydrogen bonds, which are only shown in selected images if the water is within 3.5 Å of the 10 inner glycans of the interior cellulose strands.

**Figure 15:**
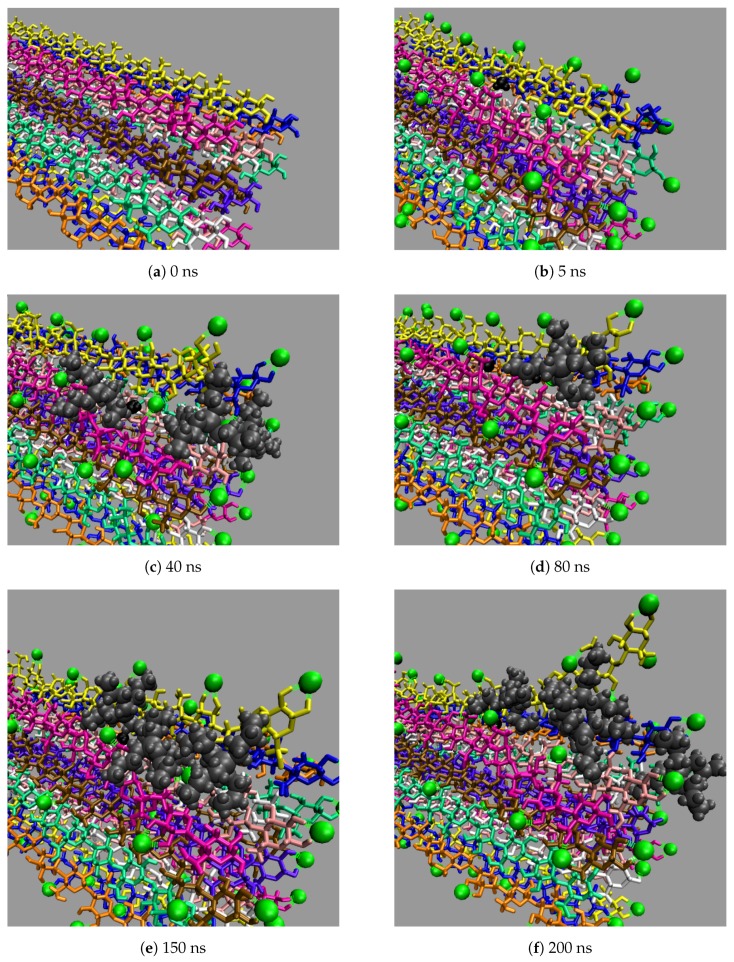
The cellulose dissolution process for the 63.1 mol % water concentration at: (**a**) 0 ns; (**b**) 5 ns; (**c**) 40 ns; (d) 80 ns; (**e**) 150 ns; and (**f**) 200 ns. The green dashed lines are the Cl–cellulose hydrogen bonds. The water–cellulose hydrogen bonds are not shown in these images.

**Figure 16:**
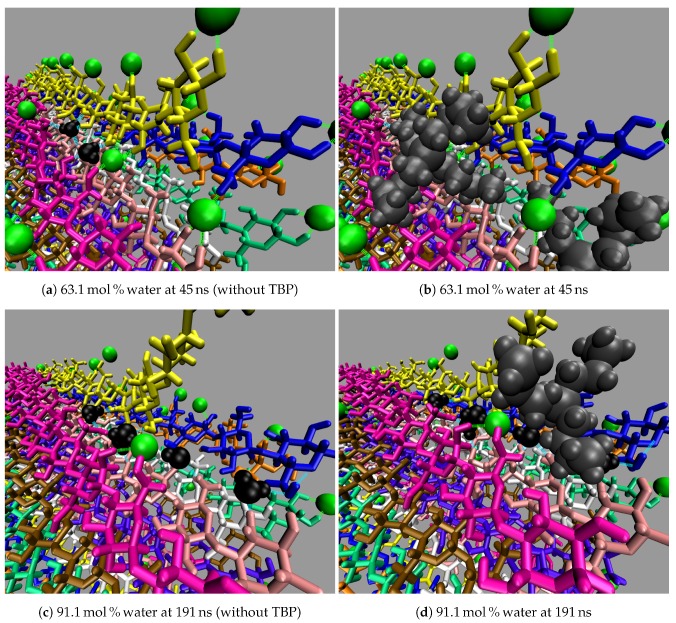
The cellulose dissolution process for: (**a**) the 63.1 mol % water concentration at 45 ns (without TBP); (**b**) the 63.1 mol % water concentration at 45 ns; (**c**) the 91.1 mol % water concentration at 191 ns (without TBP); and (**d**) the 91.1 mol % water concentration at 191 ns. The green dashed lines are the Cl–cellulose hydrogen bonds, while the light blue dashed lines are the water–cellulose hydrogen bonds.

**Figure 17:**
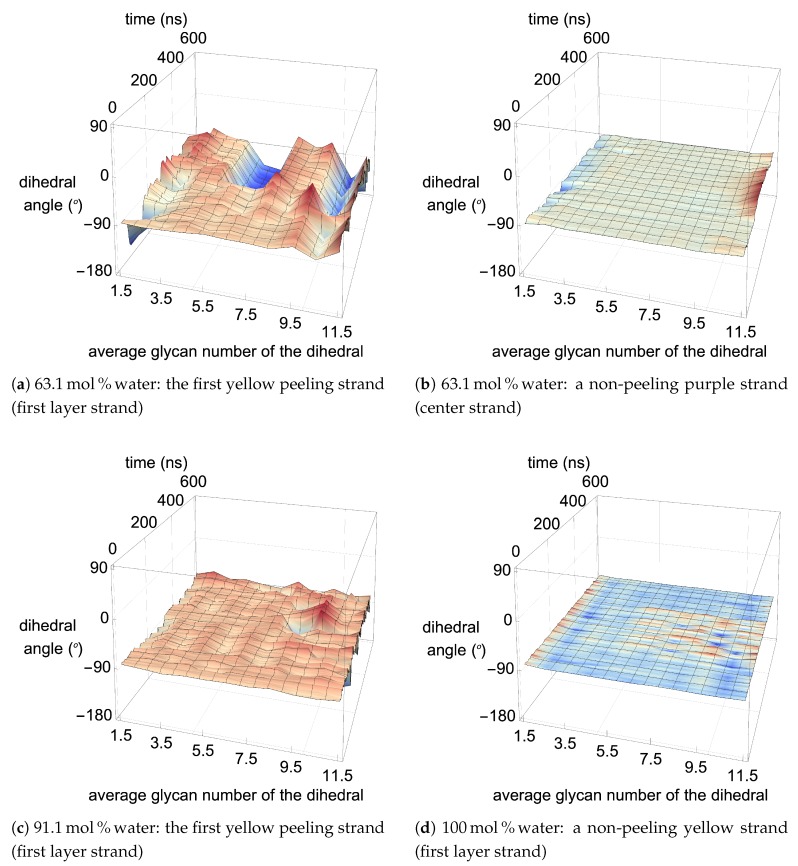
The cellulose strand twisting for various concentrations at 360 K. The cellulose strand twisting is shown between the following: (**a**) the first yellow peeling strand (first layer strand) at 63.1 mol % water; (**b**) a non-peeling purple strand (center strand) at 63.1 mol % water; (**c**) the first yellow peeling strand (first layer strand) at 91.1 mol % water; and (**d**) a non-peeling yellow strand (first layer strand) at 100 mol % water. The dihedral angle is measured, in order, from the O5-C1-O4-C4 atoms, which are between two glycans (see Figure 2). Since the dihedral angle is between two glycans in the cellulose strand, the average glycan number is used in the plots (i.e., the dihedral angle between glycans 1 and 2 is represented as 1.5). Please see Figure 4 for the cellulose strand colors. The data were averaged over 1000 data points, using a rolling average. Every 100th point was then plotted to maintain plot clarity [64].

**Figure A21:**
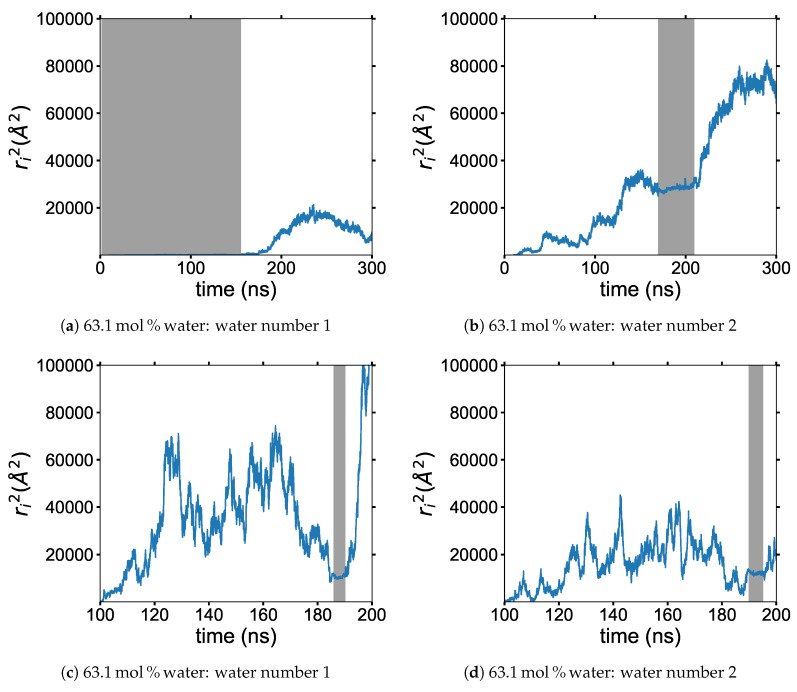
Water diffusion in and out of the pocket between the cellulose strands: (**a**) water number 1 at the 63.1 mol % water concentration; (**b**) water number 2 at the 63.1 mol % water concentration; (**c**) water number 1 at the 91.1 mol % water concentration; and (**d**) water number 2 at the 91.1 mol%water concentration. LAMMPS was utilized to calculate the squared displacement (*r*_i_^2^) of individual water molecules using the oxygen atom as the center of mass [22]. The gray shaded area indicates when the water molecule is in the pocket between the cellulose strands. The lack of variation, increase, or decrease in the squared displacement of the water molecule in the gray shaded area shows that the water molecule’s mobility can be decreased when it is in the water pocket (i.e., water’s diffusion can be decreased when the water molecules are between the cellulose strands). The water molecules show some diffusion in the gray shaded areas, some of which are attributed to the cellulose bundles movement, the water moving with the peeling cellulose strand, or the water moving in the pocket between the cellulose strands.

**Figure A22:**
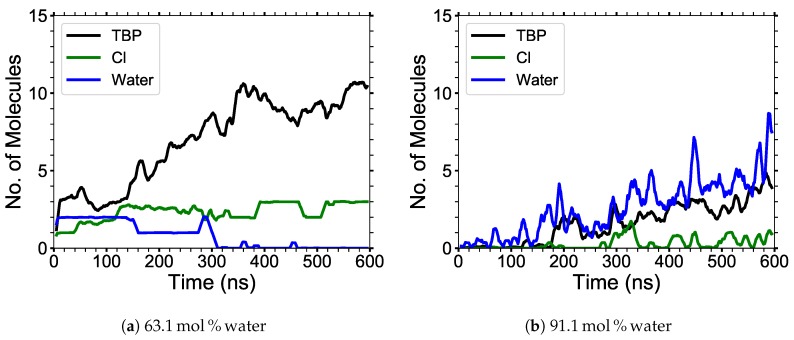
The number of molecules between the first yellow peeling strand and the cellulose bundle at various concentrations: (**a**) 63.1 mol % water; (**b**) 91.1 mol % water. The water, TBP, and Cl molecules are counted if they are within 3.5 Å of the 10 inner glycans of the light pink interior cellulose strand (see Figure 4). The data were averaged over 1000 data points, using a rolling average.

From a visual analysis of the simulations, the catalyzing anions initiate the strand peeling by disrupting the cellulose’s hydrogen bonds, which loosen the cellulose strand (see the Pairwise Energies and Hydrogen Bonding of the Peeling Strands section and Figure 11, Figure 12 and Figure 13 and Figure A11, Figure A12, Figure A13, Figure A14, Figure A15, Figure A16, Figure A17, Figure A18, Figure A19, Figure A20 for the quantitative analysis). The anion hydrogen bonding to the cellulose strands also provides a net negative pairwise energy, making the strand separation process energetically feasible. Most importantly, the anions break the celluloses intra-strand hydrogen bonds, which provides the strand with more freedom to twist and separate (see Figure 13, Figure 14, Figure 15, Figure 16 and Figure 17) [4]. The variation in the dihedral angles of the peeling strands shows that the strands are twisting throughout the dissolution process in the 63.1 mol % water and 91.1 mol % water solutions, while the same strand in the pure water solution is not twisting (see Figure 17). Since the water is adequately attracted to the cellulose’s hydroxyl groups and the anion, it inserts between the cellulose strands, ahead of the larger TBP molecule (see Table 3 and Table 4). The anions open a small pocket between the cellulose strands and remain there for a relatively long time, while the water molecules diffuse in and out of this water pocket between the cellulose strands, forming water–cellulose hydrogen bonds while in the pocket. The water molecules do insert themselves between or under the peeling strands but do not penetrate deep into the cellulose bundle. This hydrogen bonding allows the Cl and water to maintain the separation of the cellulose strand and makes the water less mobile, impeding strand reformation (see Figure 16 and Figure A21). From past research, the diffusion of the TBPCl–water solution rises with increasing water concentration [12]. Therefore, it is not surprising that the water molecules stay in the water pocket for longer durations at lower water concentrations due to the increased hydrogen bonding lifetimes and lower diffusion (see Figure A21 and Table 3 and Table 4) [12]. The chloride anion can also form multiple hydrogen bonds within or between cellulose strands, which can also act as a barrier to cellulose strand reformation. The TBP molecule pushes its way into the existing water pocket, furthering the strand separation and displacing some of the lighter water molecules. The TBP-to-water exchange ratio was determined for the 63.1 mol % water and 91.1 mol % water solutions by calculating the number of TBP or water molecules in the water pocket between the first peeling yellow strand and the light pink inner strand of the cellulose bundle (see Figure A22). The TBP-to-water exchange ratio (TBP:water) was approximately 3:1 and 1:2 for the 63.1 mol % water and 91.1 mol % water solutions, respectively. The difference in ratios is not surprising, since it is nearly identical to the ratio change in the TBPCl–water concentrations. This interaction between the TBP and the peeling strand is energetically favorable (negative) with a potential net negative pairwise energy, which likely provides a thermodynamic pathway for the cellulose separation (see Figure 11c, Figure A11c and Figure A14, Figure A15, Figure A16, Figure A17). Once the TBP molecule can insert itself between the cellulose strands, it acts as a more stable physical barrier and cleaver to separate the cellulose. The mechanism here is strikingly similar to the cellulose bundle dissolution from imidazolium-based ILs, shown by Rabideau et al. [4], although the imidazolium-based ILs were pure at higher temperatures without any co-solvent [4].

The TBP’s small tetrahedral shape is believed to play several vital roles in the ability of the TBPCl–water solution to dissolve cellulose (see Figure 14, Figure 15 and Figure 16). The first vital function of the small tetrahedral shape allows it to penetrate the small openings between the cellulose strands. The second vital function is the TBP’s shape and its ability to transform into a relatively planar form and get under the cellulose strands (i.e., the lower three arms of the TBP form a relatively planar structure), in which the rotation of the TBP also assists in the separation process (see Figure 16). The third vital function of the tetrahedral shape, quantified in our previous work [12], helps form the water vein structure, which likely shapes the TBP, Cl, and waters diffusion regime change between 8 to 92.5 mol % water [12]. The shift in the solvent’s diffusion regime raises the diffusion by approximately an order of magnitude, helping all the solvent molecules to move quickly into the openings between the cellulose strands before the strands can reform, increasing the probability of further separation [12].

## 5. Discussion

The objective of this work is to understand what hydrogen bonds, pairwise interactions, and mechanisms of the TBPCl–water mixture, or the general TBP family of ILs, are effective in cellulose dissolution, using water as a co-solvent. Using water co-solvent is crucial to the economic viability of converting biomass into biofuels, as biomass naturally contains a high mass fraction of water. Otherwise, a pre-drying step may be required before processing the biomass, or the process itself must absorb the cost of removing naturally contained water in the biomass.

The study from Wei et al. demonstrated that water could assist in cellulose dissolution by penetrating the cellulose and weakening the structure; however, the study was conducted in an alkali solution of a tetrabutylammonium hydroxide (TBAH)–urea/water system, where some reactions and deprotonations could take place [10,61,65,66,67,68]. At a water concentration where cellulose dissolution is not possible in the TBAH–water solution, 95.6 mol % water, the addition of urea likely helps to supplement for the low concentrations of TBA, which have an energetically favorable interaction with the peeling cellulose strand [10]. Furthermore, other alkali studies of LiOH-urea/water and NaOH-urea/water also suggest that water has a role in the cellulose dissolution [10,69,70]. This work builds on the analysis from Wei et al. and others, confirming that the TBPCl–water solution also exploits water to assist in the cellulose dissolution, adding mechanisms and new quantitative characteristics and thresholds.

TBPCl shows its ability to dissolve cellulose, using the co-solvent DMF, which was reported by Burns et al. (see Figure 1b) [3], who also demonstrated that the viscosity of the solution decreases with increasing DMF concentrations [3]. The TBPCl-DMF cellulose solubility profile decays with increasing water concentrations, which is quite different from the skewed Gaussian distribution profile shown from the TBPH-water solvent [2,3]. However, the TBPCl-DMF solution was tested at approximately 45 K higher temperature and only 3 data points were reported. In this work, the TBPCl–water solution also shows the cellulose solubility decaying with increasing water concentrations, but future experimental results could potentially show a maximum when the experiments are run to equilibrium, unlike these simulations. Additionally, the TBPCl–water solution could show a different cellulose solubility profile when the temperatures of the system are varied. It is also possible that the hydroxide could deprotonate the cellulose strands, or the TBPH–water solution is reacting with itself, causing the skewed Gaussian distribution profile for cellulose dissolution [61,65,66,67,68].

These simulations have shown that the anion and water can have multiple hydrogen bonds to the cellulose bundle, whether it be multiple hydrogen bonds in the same strand or between strands (see Figure 16a). Multiple hydrogen bonds were also found between acetate and the hydroxyl groups on the cellulose, in the tetrabutylammonium acetate (TBAAc) dimethyl sulfoxide (DMSO) solution, but the TBA cations do not show the same type of dual bonding [60]. This work shows similar results, as the TBP cation and water have very low hydrogen bonding strength or hydrogen bonding lifetimes with cellulose when compared to the anions.

The intra-strand, inter-strand, and solvent hydrogen bonding were compared at various IL–water concentrations between a small and large Iβ cellulose bundle (18 vs. 88 strands, respectively). The intra-strand hydrogen bonding for the solvent-exposed strands was nearly the same between the small and large Iβ cellulose bundles. The center strands in the small bundle had significantly less intra-strand hydrogen bonds, due to less bundle stabilization from the other cellulose strands when compared to the large cellulose bundle. This is an important result, as the solvent-exposed strands of the small and large bundles must have matching intra-strand hydrogen bonds, since breaking them is the critical first step in the IL-based cellulose dissolution process. The inter-strand bonding was significantly different between the small and large bundles, except for the 63.1 mol % water concentration, where the large bundle was nearly identical to the small bundle’s inter-strand bonding. However, more research is required to determine precisely why the inter-strand hydrogen bonding is the same at 63.1 mol % water but differs for all the higher concentrations. The nearly identical inter-strand hydrogen bonding at 63.1 mol % water could be an artifact of the GLYCAM06 force field with the low dispersion and Coulombic forces near the cellulose at the start of the simulations (i.e., the void space of at least 2 Å from the cellulose bundles at the start of the simulation) [19,31]. The TBPCl–water solvent’s hydrogen bonding with the cellulose strands was nearly the same for the small and large cellulose bundles. Overall, the small bundle’s solvent-exposed strands appear to be a fair representation of the large cellulose bundle in the TBPCl–water system. For many similar cases, the small bundle may be an adequate replacement for the larger cellulose bundle, while significantly minimizing the computational expense of the simulations.

**Figure 18:**
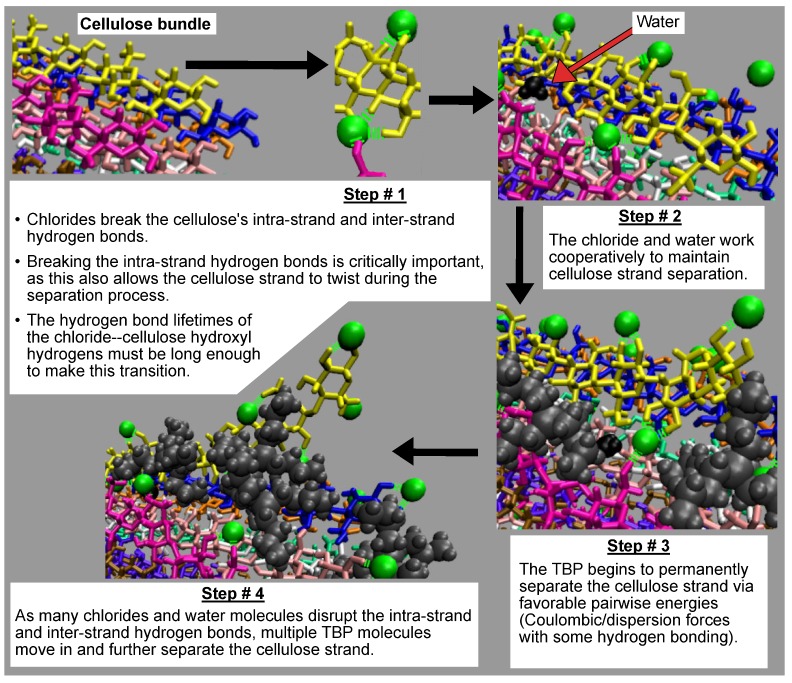
Summarized mechanism for cellulose dissolution in the TBPCl–water solution. These simulation snapshots are from the 63.1 mol % water simulation. The green dashed lines indicate the chloride–cellulose hydrogen bonds. The water hydrogen bonds are not shown in this Figure.

The cellulose dissolution mechanism for the TBPCl–water solution is broken down into four key steps and summarized in Figure 18. In step one, the chlorides must break the intra-strand hydrogen bonds (O5⋯HO3-O3 and O6⋯HO2-O2), allowing the cellulose to twist freely at the glycan connection point (i.e., 1,4 location/O5-C1-O4-C4 dihedral angle) [4]. A single chloride is hydrogen-bonded to both intra-strand hydrogen bonding locations, Cl⋯O2-HO2 and Cl⋯O3-HO3, at the same time (see the step #1 image: the bottom hydrogen bonding in the yellow strand). The chlorides are very capable of breaking the intra-strand hydrogen bonds until the water concentration gets too high, around 80 mol % water. Beyond 80 mol % water, the Cl⋯HO2-O2 and Cl⋯HO3-O3 hydrogen bonding lifetimes fall below the intra-strand hydrogen lifetimes, reducing the probability of sustained intra-strand hydrogen bonding disruption (see Figure 10). The increased diffusion of the TBPCl–water solution above 80 mol % water may slow the rapid decline of the cellulose dissolution [12]. The chlorides can also disrupt the inter-strand hydrogen bonds, disconnecting the strand from its neighboring strand. The chloride anions have a net negative pairwise interaction with the cellulose strand, while being able to hold multiple hydrogen bonds with the cellulose strand/bundle. The net negative pairwise interaction of the chloride offsets the net positive pairwise interaction of the peeling cellulose strand, making the process more thermodynamically feasible [4]. In step two, the chlorides and water form small gaps between the strand and its neighboring strands via physical separation and hydrogen bonding. These small gaps delay the reformation of the cellulose strand, so the TBP cation has time to finish the strand separation before the cellulose strand reforms. Water is capable of hydrogen bonding to the cellulose strand for shorter durations. The water hydrogen bonds are not shown in Figure 18, but are shown in Figure 16. In step three, the TBP moves in and further and more permanently separates the cellulose strand. The Coulombic and dispersion forces, hydrogen bonding, and the shape of the TBP molecule allow it to separate the cellulose strand effectively. Additionally, the TBP cations, at worst, have an overall net neutral pairwise interaction with the cellulose strand, and a favorable pairwise interaction with the peeling cellulose strand. A unique attribute of the TBP cation when compared to the imidazolium cation is the consistently favorable (negative) pairwise energy throughout the entire dissolution process [4]. In step four, more TBP molecules move in and separate the strand further. At the same time, the chloride hydrogen bonding is still providing flexibility to the separating strand, while again using the attributes of steps one, two, and three.

## 6. Conclusions

The TBPCl–water solution has demonstrated its potential for dissolving cellulose, and this study identified some hydrogen bonds, pairwise interactions, and mechanisms that drive the process. The water, Cl, and TBP molecules all work together as a cooperative mechanism in the cellulose dissolution process until the solution becomes too diluted with water. By stark contrast, many other ionic liquids do not work well at low to moderate temperatures while in the presence of water [1,5,6,7,9]. This study shows a common theme of anions loosening the hydrogen bonds of the cellulose strands and water partially maintaining strand separation while the cations cleave the strands away from the bundle [3,4,9,13,60,61,71,72]. In the TBPCl–water solution, the chloride anions have strong hydrogen bonding lifetimes with cellulose (Cl⋯HO2-O2, Cl⋯HO3-O3, and Cl⋯HO6-O6), and are capable of holding multiple hydrogen bonds with the cellulose strands. The anion helps to cleave the strands by opening them up and allowing water and TBP to assist in the dilution process [3,4,9,13,60,61,71,72]. While chloride anions remain near or between the cellulose strands by hydrogen-bonding to them, the moderate strength of the Cl⋯Hw-Ow, Ow⋯HO2-O2, and Ow⋯HO6-O6 hydrogen bonding pairs attracts water to the local area, inhibiting the reformation of the cellulose strands via the water-cellulose hydrogen bonding and physical impedance. These localized anion/water pockets provide a buffer time for the TBP molecules to enter and assist with cleaving the cellulose strands. As the cellulose dissolution begins to rapidly plummet between 79.4 to 86.8 mol % water, the Cl⋯HO2-O2 and Cl⋯HO3-O3 hydrogen bond lifetimes both fall below the largest of the intra-strand hydrogen bonding lifetimes (O5⋯HO3-O3 and O6⋯HO2-O2). Specifically, the hydrogen bonding lifetimes in the Cl⋯Hw-Ow, Cl⋯HO2-O2, and Cl⋯HO3-O3 pairs, between 63.1 to 86.8 mol % water, decay by roughly 0.8, 1.6, and 2.1 orders of magnitude, respectively. However, we believe that the diffusion regime shift in this region, between 80 to 92.5 mol % water [12], helps to subsidize these losses in the hydrogen-bonding lifetime and impede the decay of cellulose dissolution at these high water concentrations. The anion–peeling strand net negative pairwise energies offset the bundle–peeling strand net positive pairwise energies [4]. The TBP–cellulose pairwise energy is favorable and at least overall net neutral, and may even have a slight net negative energy during and after the cellulose peeling process. When the cellulose strand peeling stops, TBP–peeling cellulose strand pairwise energy is relaxed back to its initial or slightly lower pairwise energy. This unique and favorable pairwise energy between the TBP and the peeling cellulose strand, during its peeling process, could be a unique characteristic of the TBPCl–water solution and TBP family of ILs.

## Figures and Tables

**Table 1:** Small cellulose bundle (18 strands) simulation compositions.

Mol % Water	Total Atoms	Cellulose Atoms	Total Solvent Atoms	Cl Molecules	TBP Molecules	Water Molecules
63.1	95,058	4590	90,468	1530	1530	2616
79.4	95,262	4590	90,672	1383	1383	5330
86.8	95,199	4590	90,609	1229	1229	8081
91.1	95,565	4590	90,975	1074	1074	10,993
93.9	96,054	4590	91,464	913	913	14,054
95.8	96,288	4590	91,698	749	749	17,084
100	108,507	4590	103,917	0	0	34,639

**Table 2:** Large cellulose bundle (88 strands) simulation compositions.

Mol % Water	Total Atoms	Cellulose Atoms	Total Solvent Atoms	Cl Molecules	TBP Molecules	Water Molecules
63.1	625,626	44,616	581,010	9826	9826	16,802
79.4	621,438	44,616	576,822	8798	8798	33,910
86.8	624,333	44,616	579,717	7863	7863	51,705
91.1	618,765	44,616	574,149	6778	6778	69,379
93.9	617,445	44,616	572,829	5718	5718	88,019
95.8	614,886	44,616	570,270	4658	4658	106,246
100	659,613	44,616	614,997	0	0	204,999

**Table 3:** TBPCl hydrogen bonding lifetimes (ps), Part 1 of 5.

A⋯H-D ${}^{a}$	mol % Water						
	63.1	79.4	86.8	91.1	93.9	95.8	100
Cl⋯H’s-C’s ${}^{b}$ (10 ns)	1.06	0.683	0.592	0.598	0.365	0.728	−−
Cl⋯H’s-C’s ${}^{b}$ (400 ns)	0.543	0.654	1.13	0.802	0.644	0.490	−−
Cl⋯H’s-CT’s ${}^{b,d}$ (10 ns)	0.158	0.152	0.147	0.147	0.144	0.143	−−
Cl⋯H’s-CT’s ${}^{b,d}$ (400 ns)	0.160	0.151	0.146	0.148	0.140	0.145	−−
Cl⋯HO2-O2 (10 ns)	27,329	7370	658	109	54.3	48.9	−−
Cl⋯HO2-O2 (400 ns)	>31,445	8615	1184	346	104	51.6	−−
Cl⋯HO3-O3 (10 ns)	19,816	1197	147	55.2	11.7	31.0	−−
Cl⋯HO3-O3 (400 ns)	>20,848	>5941	80.2	203	32.6	8.20	−−
Cl⋯HO6-O6 (10 ns)	7189	422	63.4	52.3	9.33	6.78	−−
Cl⋯HO6-O6 (400 ns)	6487	366	45.5	9.78	13.4	11.3	−−
Cl⋯Hw-Ow (10 ns)	21.0	7.04	2.62	2.05	1.77	1.65	−−
Cl⋯Hw-Ow (400 ns)	20.8	7.10	4.14	2.05	1.77	1.67	−−
CT⋯H’s-C’s ${}^{b}$ (10 ns)	0.396	0.375	0.361	0.320	0.285	0.297	−−
CT⋯H’s-C’s ${}^{b}$ (400 ns)	0.427	0.374	0.316	0.326	0.328	0.297	−−
CT⋯HO’s-O’s ${}^{b}$ (10 ns)	0.076	0.089	0.150	0.119	0.158	0.123	−−
CT⋯HO’s-O’s ${}^{b}$ (400 ns)	0.138	0.072	0.137	0.103	0.080	0.093	−−
CT⋯Hw-Ow (10 ns)	0.038	0.033	0.030	0.029	0.027	0.026	−−
CT⋯Hw-Ow (400 ns)	0.037	0.033	0.030	0.028	0.028	0.026	−−

${}^{a}$ A⋯H-D = hydrogen Acceptor atom⋯Hydrogen atom–hydrogen Donor atom. Note: (H-D) share a covalent bond. Data was started at 10.2 and 400.2 ns. ${}^{b}$ Averaged data ${}^{c}$ NA means no hydrogen bonds found at the start of these calculations. ${}^{d}$ The H’s are the HP and HC2, HC3, and HC4 atoms in the TBP molecule. ${}^{f}$ MDAnalysis H-bond lifetimes do not work with >100,000 atoms in the analysis. For lifetimes without auto-correlation values of zero (i.e., values with tha > symbol), the final auto-correlation values are <0.05 unless otherwise noted: ${}^{g}$ <0.1; ${}^{h}$ <0.15.

**Table 4:** TBPCl hydrogen bonding lifetimes (ps), Part 2 of 5.

A⋯H-D ${}^{a}$	mol % Water						
	63.1	79.4	86.8	91.1	93.9	95.8	100
Ow⋯H’s-C’s ${}^{b}$ (10 ns)	0.723	0.693	0.370	0.464	0.405	0.394	0.325
Ow⋯H’s-C’s ${}^{b}$ (400 ns)	NA ${}^{c}$	0.451	0.621	0.468	0.380	0.419	0.325
Ow⋯H’s-CT’s ${}^{b,d}$ (10 ns)	0.159	0.155	0.154	0.150	0.148	0.148	−−
Ow⋯H’s-CT’s ${}^{b,d}$ (400 ns)	0.159	0.158	0.154	0.152	0.149	0.146	−−
Ow⋯HO2-O2 (10 ns)	11.3	8.08	4.92	4.12	1.94	2.87	1.10
Ow⋯HO2-O2 (400 ns)	NA ${}^{c}$	1.79	6.00	2.72	3.40	2.52	1.05
Ow⋯HO3-O3 (10 ns)	0.026	1.08	0.584	0.917	0.747	0.749	0.596
Ow⋯HO3-O3 (400 ns)	NA ${}^{c}$	NA ${}^{c}$	0.617	0.731	1.23	2.58	0.611
Ow⋯HO6-O6 (10 ns)	6.63	12.1	4.19	3.03	3.20	2.34	0.792
Ow⋯HO6-O6 (400 ns)	NA ${}^{c}$	5.02	3.81	3.28	3.64	2.80	0.789
O2⋯H’s-CT’s ${}^{b,d}$ (10 ns)	0.207	0.173	0.196	0.210	0.180	0.175	−−
O2⋯H’s-CT’s ${}^{b,d}$ (400 ns)	0.202	0.199	0.194	0.185	0.199	0.176	−−
O3⋯H’s-CT’s ${}^{b,d}$ (10 ns)	0.210	0.177	0.171	0.186	0.215	0.181	−−
O3⋯H’s-CT’s’s ${}^{b,d}$ (400 ns)	0.215	0.191	0.178	0.164	0.181	0.181	−−
O6⋯H’s-CT’s ${}^{b,d}$ (10 ns)	0.206	0.210	0.190	0.207	0.176	0.186	−−
O6⋯H’s-CT’s ${}^{b,d}$ (400 ns)	0.220	0.203	0.194	0.195	0.197	0.189	−−
O4⋯H’s-CT’s ${}^{b,d}$ (10 ns)	0.222	0.204	0.176	0.199	0.161	0.181	−−
O4⋯H’s-CT’s ${}^{b,d}$ (400 ns)	0.214	0.202	0.246	0.181	0.176	0.186	−−
O5⋯H’s-CT’s ${}^{b,d}$ (10 ns)	0.236	0.207	0.198	0.178	0.183	0.199	−−
O5⋯H’s-CT’s ${}^{b,d}$ (400 ns)	0.200	0.223	0.205	0.207	0.166	0.179	−−
O5⋯HO3-O3 (10 ns)	15.3	17.4	17.4	18.8	16.9	4.54	3.85
O5⋯HO3-O3 (400 ns)	3.26	3.12	11.9	14.4	13.3	2.88	11.8
O6⋯HO2-O2 (10 ns)	3009	428	497	2,423	2,830	2327	588
O6⋯HO2-O2 (400 ns)	457	62.6	388	505	3318	2082	2066

${}^{a}$ A⋯H-D = hydrogen Acceptor atom⋯Hydrogen atom–hydrogen Donor atom. Note: (H-D) share a covalent bond. Data was started at 10.2 and 400.2 ns. ${}^{b}$ Averaged data ${}^{c}$ NA means no hydrogen bonds found at the start of these calculations. ${}^{d}$ The H’s are the HP and HC2, HC3, and HC4 atoms in the TBP molecule. ${}^{f}$ MDAnalysis H-bond lifetimes do not work with >100,000 atoms in the analysis. For lifetimes without auto-correlation values of zero (i.e., values with the > symbol), the final auto-correlation values are <0.05 unless otherwise noted: ${}^{g}$ <0.1; ${}^{h}$ <0.15.

## Abbreviations

The following abbreviations are used in this manuscript:

Ac	Acetate
A⋯H-D	hydrogen Acceptor atom⋯Hydrogen atom–hydrogen Donor atom
AMBER	Assisted Model Building with Energy Refinement
AMIMCl	1-allyl-3-methylimidazolium chloride
C	Carbon
Cx	Carbon of location number x in the cellulose strand
CTx	Carbon of location number x in the TBP molecule
Cl	Chloride
DMF	Dimethylformamide
DMSO	Dimethylsulfoxide
EMIM	1-ethyl-3-methylimidazolium
GLYCAM06	Glycosylation-dependent Cell Adhesion Molecule 2006
H	Hydrogen
Hx	Hydrogen of location number x in the cellulose strand
HCx	Hydrogen connected to CTx of the TBP molecule (x only ranges from 2 to 4)
HP	Hydrogen connected to CT1 of the TBP molecule
Hw	Hydrogen of water
IL	Ionic Liquid
LAMMPS	Large-scale Atomic/Molecular Massively Parallel Simulator
LJ	Lennard-Jones
Li	Lithium
MD	Molecular Dynamics
Na	Sodium
NPT	Isobaric-isothermal
NVT	Constant volume-isothermal
O	Oxygen
Ox	Oxygen of location number x in the cellulose strand
OH	Hydroxide or hydroxyl group
Ow	Oxygen of water
P	Phosphorous
PPPM	Particle-Particle Particle-Mesh
TBA	Tetrabutylammonium
TBAH	Tetrabutylammonium hydroxide
TBP	Tetrabutylphosphonium
TBPCl	Tetrabutylphosphonium chloride
TBPH	Tetrabutylphosphonium hydroxide
TIP3P	Three-Site Transferrable Intermolecular Potential
VDW	Van der Waals
VMD	Visual Molecular Dynamics

## Appendix A

Additional data are provided for the small vs. large cellulose bundle comparison, which includes the standard deviations of the averaged data over 10 to 20 ns. Additional data are provided for the small Iβ cellulose bundle dissolution in the TBPCl–water simulations to paint a clear picture of the whole dissolution process. The cellulose dissolution is provided in the number of dissolved glycans. The additional cellulose dissolution rates, images, pairwise energies and hydrogen bonding of the individual separating yellow strands or non-separating yellow strands are provided below. Several other hydrogen bonding lifetimes were also calculated in this study and are included, primarily including the lifetimes between the cellulose bundle itself. The diffusion of singular water molecules and the number of molecules between the peeling strand and an inner strand of the cellulose bundle are also provided.

### Appendix A.2. The Extent of Cellulose Dissolution

#### Cellulose Dissolution

**Table A1:** Small cellulose bundle dissolution rates

Mol %	Cellulose Dissolution Rate
Water	(wt % /ns)
63.1	1.443 × $10^{-3}$
79.4	1.505 × $10^{-3}$
86.8	0.501 × $10^{-3}$
91.1	0.326 × $10^{-3}$
93.9	0.033 × $10^{-3}$
95.8	0.000 × $10^{-3}$
100	0.000 × $10^{-3}$

### Appendix A.4. Hydrogen Bonding Lifetimes

**Table A2:** TBPCl hydrogen bonding lifetimes (ps), Part 3 of 5.

A⋯H-D ${}^{a}$	mol % Water						
	63.1	79.4	86.8	91.1	93.9	95.8	100
O2⋯H1-C1 (10 ns)	16.4	103	105	25.8	18.4	8.62	3.51
O2⋯H1-C1 (400 ns)	13.9	33.1	171	14.0	12.7	146	24.7
O2⋯H2-C2 (10 ns)	0.290	1.03	0.196	0.241	0.247	0.244	0.248
O2⋯H2-C2 (400 ns)	0.907	0.222	0.433	0.339	0.554	0.322	0.247
O2⋯H3-C3 (10 ns)	1.86	2.37	1.88	2.03	1.98	2.04	2.44
O2⋯H3-C3 (400 ns)	1.10	2.14	1.68	1.80	1.73	2.16	1.67
O2⋯H4-C4 (10 ns)	3661	3077	3304	2230	2039	4170	414
O2⋯H4-C4 (400 ns)	49.8	386	2598	415	419	492	2193
O2⋯H5-C5 (10 ns)	0.679	0.359	0.166	0.242	0.301	0.183	0.237
O2⋯H5-C5 (400 ns)	1.22	0.766	0.176	0.234	0.682	0.215	0.324
O2⋯H6’s-C6 ${}^{b}$ (10 ns)	0.589	0.588	0.505	0.545	0.480	0.381	0.410
O2⋯H6’s-C6 ${}^{b}$ (400 ns)	0.541	0.680	0.619	0.600	0.588	0.530	0.465
O3⋯H1-C1 (10 ns)	1,703	14.9	22.6	4.05	16.2	12.7	11.8
O3⋯H1-C1(400 ns)	213	39.6	33.8	193	148	24.5	4.27
O3⋯H2-C2 (10 ns)	237	36.2	38.0	34.5	40.6	46.0	41.3
O3⋯H2-C2 (400 ns)	41.1	35.7	23.4	32.2	29.5	32.3	33.3
O3⋯H3-C3 (10 ns)	0.948	0.701	0.662	0.934	1.01	0.771	0.843
O3⋯H3-C3(400 ns)	0.628	0.836	0.978	0.807	0.953	0.726	0.657
O3⋯H4-C4 (10 ns)	1.23	0.405	0.234	0.402	0.977	0.240	0.316
O3⋯H4-C4 (400 ns)	0.262	0.571	1.16	1.20	0.473	0.166	0.292
O3⋯H5-C5 (10 ns)	278	223	2812	2337	2924	34.4	9.23
O3⋯H5-C5 (400 ns)	4364	321	1618	401	>5210	37.5	1455
O3⋯H6’s-C6 ${}^{b}$ (10 ns)	0.119	0.144	0.134	0.113	0.187	0.107	0.114
O3⋯H6’s-C6 ${}^{b}$ (400 ns)	0.187	0.216	0.232	0.193	0.196	0.160	0.140

${}^{a}$ A⋯H-D = hydrogen Acceptor atom⋯Hydrogen atom–hydrogen Donor atom. Note: (H-D) share a covalent bond. Data was started at 10.2 and 400.2 ns. ${}^{b}$ Averaged data ${}^{c}$ NA means no hydrogen bonds found at the start of these calculations. ${}^{d}$ The H’s are the HP and HC2, HC3, and HC4 atoms in the TBP molecule. ${}^{f}$ MDAnalysis H-bond lifetimes do not work with >100,000 atoms in the analysis. For lifetimes without auto-correlation values of zero (i.e., values with > symbol), the final auto-correlation values are <0.05 unless otherwise noted: ${}^{g}$ <0.1; ${}^{h}$ <0.15.

**Table A3:** TBPCl hydrogen bonding lifetimes (ps), Part 4 of 5.

A⋯H-D ${}^{a}$	mol % Water						
	63.1	79.4	86.8	91.1	93.9	95.8	100
O4⋯H1-C1 (10 ns)	NA ${}^{c}$	NA ${}^{c}$	NA ${}^{c}$	NA ${}^{c}$	NA ${}^{c}$	NA ${}^{c}$	NA ${}^{c}$
O4⋯H1-C1 (400 ns)	NA ${}^{c}$	1.35	NA ${}^{c}$	0.137		NA ${}^{c}$	NA ${}^{c}$
O4⋯H2-C2 (10 ns)	>50,596	>48,068	20,473	11,651	10,386	29,122	4834
O4⋯H2-C2 (400 ns)	>57,152 ${}^{h}$	>29,957 ${}^{g}$	>13,465	>13,689	15,605	>24,677	5030
O4⋯H3-C3 (10 ns)	NA ${}^{c}$	0.609	NA ${}^{c}$	NA ${}^{c}$	NA ${}^{c}$	NA ${}^{c}$	NA ${}^{c}$
O4⋯H3-C3 (400 ns)	1154	3140	8.09	6.13	7.71	NA ${}^{c}$	1.45
O4⋯H4-C4 (10 ns)	NA ${}^{c}$	0.126	0.243	NA ${}^{c}$	NA ${}^{c}$	NA ${}^{c}$	0.114
O4⋯H4-C4 (400 ns)	0.201	0.139	0.266	0.304	2.65	NA ${}^{c}$	0.306
O4⋯H5-C5 (10 ns)	5.53	4.53	0.753	0.771	0.939	0.637	0.591
O4⋯H5-C5 (400 ns)	8.43	932	2.58	80.8	137	0.201	2.41
O4⋯H6’s-C6 ${}^{b}$ (10 ns)	0.096	0.096	0.076	0.065	0.068	0.086	0.099
O4⋯H6’s-C6 ${}^{b}$ (400 ns)	0.414	0.223	0.529	0.106	0.106	0.074	0.064
O5⋯H1-C1 (10 ns)	NA ${}^{c}$	17.7	NA ${}^{c}$	5.19	NA${}^{c}$	NA ${}^{c}$	NA ${}^{c}$
O5⋯H1-C1 (400 ns)	NA ${}^{c}$	NA ${}^{c}$	NA ${}^{c}$	NA ${}^{c}$	NA ${}^{c}$	NA ${}^{c}$	NA ${}^{c}$
O5⋯H2-C2 (10 ns)	0.287	0.085	0.057	0.073	0.196	0.127	0.139
O5⋯H2-C2 (400 ns)	0.077	0.314	0.384	0.223	2.44	0.065	0.109
O5⋯H3-C3 (10 ns)	0.308	0.500	0.438	0.456	0.312	0.442	0.512
O5⋯H3-C3 (400 ns)	0.951	2.12	0.464	0.351	0.478	0.396	0.541
O5⋯H4-C4 (10 ns)	1.78	0.501	NA ${}^{c}$	NA ${}^{c}$	NA ${}^{c}$	NA ${}^{c}$	NA ${}^{c}$
O5⋯H4-C4 (400 ns)	1,612	9.91	8.62	NA ${}^{c}$	0.241	NA ${}^{c}$	NA ${}^{c}$
O5⋯H5-C5 (10 ns)	0.410	0.377	0.365	0.504	0.816	0.364	0.350
O5⋯H5-C5 (400 ns)	9.15	0.894	0.287	0.325	1.51	0.316	0.354
O5⋯H6’s-C6 ${}^{b}$ (10 ns)	0.360	0.459	0.460	0.403	0.364	0.375	0.352
O5⋯H6’s-C6 ${}^{b}$ (400 ns)	0.498	0.596	0.576	0.366	0.312	0.447	0.402

*^a^* A⋯H-D = hydrogen Acceptor atom⋯Hydrogen atom–hydrogen Donor atom. Note: (H-D) share a covalent bond. Data was started at 10.2 and 400.2 ns. *^b^* Averaged data *^c^* NA means no hydrogen bonds found at the start of these calculations. *^d^* The H’s are the HP and HC2, HC3, and HC4 atoms in the TBP molecule. *^f^* MDAnalysis H-bond lifetimes do not work with >100,000 atoms in the analysis. For lifetimes without auto-correlation values of zero (i.e., values with > symbol), the final auto-correlation values are <0.05 unless otherwise noted: *^g^* <0.1; *^h^* <0.15.

**Table A4:** TBPCl hydrogen bonding lifetimes (ps), Part 5 of 5.

A⋯H-D ${}^{a}$	mol % Water						
	63.1	79.4	86.8	91.1	93.9	95.8	100
O6⋯H1-C1 (10 ns)	11.9	31.7	0.922	0.866	0.759	0.500	0.663
O6⋯H1-C1 (400 ns)	5.91	74.9	4.51	0.826	4.83	1.03	3.87
O6⋯H2-C2 (10 ns)	0.268	0.317	0.217	0.252	0.337	0.231	0.334
O6⋯H2-C2 (400 ns)	0.424	0.552	0.797	0.531	0.641	0.416	0.433
O6⋯H3-C3 (10 ns)	0.758	0.351	0.695	0.722	0.717	0.258	0.557
O6⋯H3-C3 (400 ns)	1.51	8.21	0.858	1.42	38.6	0.962	0.665
O6⋯H4-C4 (10 ns)	0.801	0.828	0.269	0.348	0.420	0.238	0.359
O6⋯H4-C4 (400 ns)	4.94	0.717	1.03	0.417	0.686	0.323	1.15
O6⋯H5-C5 (10 ns)	16.2	4.00	4.41	17.2	5.35	3.97	10.8
O6⋯H5-C5 (400 ns)	15.5	2.11	2.68	2.47	3.40	6.69	4.70
O6⋯H6’s-C6 ${}^{b}$ (10 ns)	0.249	0.201	0.202	0.211	0.204	0.122	0.172
O6⋯H6’s-C6 ${}^{b}$ (400 ns)	0.343	0.370	0.271	0.434	0.316	0.216	0.275
Ow⋯Hw-Ow (10 ns)	0.227	0.429	0.548	0.555	0.526	0.484	NA ${}^{c,f}$
Ow⋯Hw-Ow (400 ns)	0.239	0.436	0.555	0.558	0.525	0.487	NA ${}^{c,f}$

${}^{a}$ A⋯H-D = hydrogen Acceptor atom⋯Hydrogen atom–hydrogen Donor atom. Note: (H-D) share a covalent bond. Data was started at 10.2 and 400.2 ns. ${}^{b}$ Averaged data ${}^{c}$ NA means no hydrogen bonds found at the start of these calculations. ${}^{d}$ The H’s are the HP and HC2, HC3, and HC4 atoms in the TBP molecule. ${}^{f}$ MDAnalysis H-bond lifetimes do not work with >100,000 atoms in the analysis. For lifetimes without auto-correlation values of zero (i.e., values with > symbol), the final auto-correlation values are <0.05 unless otherwise noted: ${}^{g}$ <0.1; ${}^{h}$ <0.15.
